# Adenosine and guanosine-based oligonucleotides-loaded PLGA nanoparticles attenuates progression of surgically induced osteoarthritis

**DOI:** 10.1007/s13346-025-02020-6

**Published:** 2025-12-15

**Authors:** Yoonhee Kim, Jin Han, Ji Young Park, Seungwoo Han

**Affiliations:** 1 Department of Biomedical Science, The Graduate School, Kyungpook National University, Daegu, Republic of Korea; 2 Division of Rheumatology, Department of Internal Medicine, School of Medicine, Kyungpook National University, 130 Dongdeok-ro, Jung-gu, Daegu, 41921 Republic of Korea; 3 Department of Pathology, School of Medicine, Case Western Reserve University, Cleveland, OH United States of America; 4 Department of Pathology, School of Medicine, Kyungpook National University, Daegu, Republic of Korea

**Keywords:** Osteoarthritis, Purinergic signaling, Adenosine, Guanosine, PLGA nanoparticles

## Abstract

Osteoarthritis (OA) is a chronic degenerative joint disease that lacks effective therapies to halt its progression. While endogenous purinergic signaling—particularly via adenosine—shows promise for reducing inflammation, it is limited by short half-life and off-target effects. To address these limitations, we developed an optimal anti-inflammatory adenosine-guanosine-based oligonucleotide encapsulated in poly(lactic-co-glycolic) acid (PLGA)-based nanoparticles (NanoOligo) to enhance in vivo stability and investigated its impact on surgically induced OA models and the underlying mechanisms responsible for its anabolic effects. A large oligonucleotide library (482 unique 10- to 20-mer sequences) was screened in RAW264.7 macrophages under LPS-induced inflammation to identify the most potent candidate, which was then encapsulated into PLGA nanoparticles using a microfluidic system. NanoOligo significantly protected against cartilage degeneration and alleviated pain behaviors in the rat ACLT + pMx model following intra-articular administration. In IL-1β–treated chondrocytes, it markedly suppressed inflammatory cytokines (TNFα, IL-6) and catabolic proteases (MMP-3, MMP-13, ADAMTS5). Mechanistically, NanoOligo’s anti-catabolic effects were dependent on A1R and A2AR, leading to activation of the PKA–CREB axis and suppression of p38 MAPK signaling, which in turn reduced oxidative stress and cellular senescence via upregulation of the Sirt1–Nrf2–HO-1 antioxidant pathway. Collectively, these findings support joint-localized purinergic modulation as a potential therapeutic target for OA treatment, aligning structural protection with improvements in pain-related behaviors.

**Graphical abstract:**

Schematic of the proposed mechanism of action for the adenosine–guanosine-based oligonucleotide, via the A1R- and A2AR-mediated PKA–CREB axis and Sirt1–Nrf2–HO-1 pathway.

The oligonucleotide is thought to release adenosine (Ad) and guanosine (Gu) moieties that bind to A1R and A2AR on the cell surface. Receptor activation increases intracellular cAMP levels, thereby stimulating PKA. Subsequently, the Sirt1–Nrf2–HO-1 and CREB antioxidant pathways are activated, reducing ROS levels. This decline in ROS feeds back to prematurely terminate p38 MAPK activation, a key driver of catabolic processes in OA. As a result, MMP-13 expression is diminished, ultimately preserving extracellular matrix integrity.

**Supplementary Information:**

The online version contains supplementary material available at 10.1007/s13346-025-02020-6.

## Highlights

A potent anti-inflammatory adenosine–guanosine-based oligonucleotide was identified by screening a large library about 500 sequences.

Encapsulation within PLGA nanoparticles (NanoOligo) improved in vivo stability and delivery to chondrocytes.

NanoOligo significantly reduced inflammatory and catabolic mediators while protecting cartilage from degeneration in a rat ACLT + pMx model as well as mice DMM model.

Its therapeutic efficacy was mediated through A1R- and A2AR-dependent PKA–CREB activation and upregulation of the Sirt1–Nrf2–HO-1 antioxidant pathway.

**Supplementary Information:**

The online version contains supplementary material available at 10.1007/s13346-025-02020-6.

## Graphical abstract

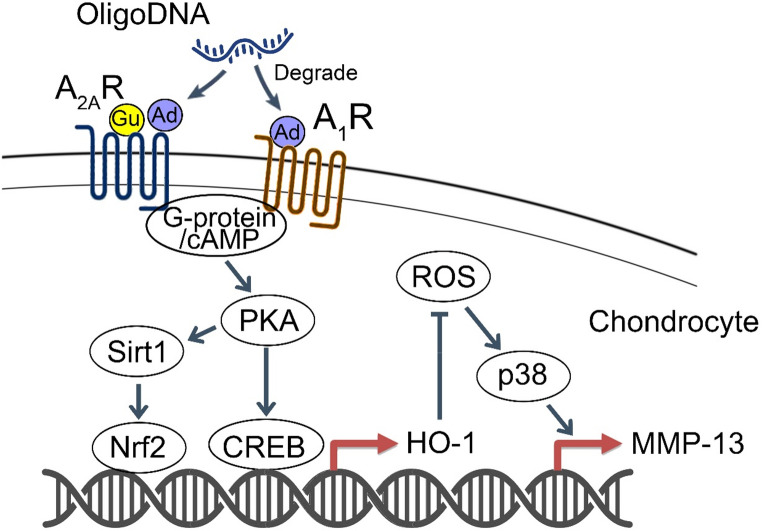

## Introduction

Osteoarthritis (OA) is the most prevalent degenerative joint disorder, leading to significant disability, yet there is no treatment modality that slows its progression [1]. Until now, the main treatment target for OA has been limited to pain control [2]. NSAIDs are used as first line treatment for pain control, and in cases of persistent pain that NSAIDs cannot alleviate, intra-articular steroid injection is recommended [2]. However, the cardiovascular and gastrointestinal risks of NSAIDs are well-documented, and the effects of steroid injections are only temporary [3, 4]. Moreover, recent studies have revealed that repetitive steroid injections may even hasten the joint space narrowing of knee, casting doubt on their safety [4, 5]. Given these concerns, the pressing need for safer pain management options is evident, but the development of alternatives to NSAIDs and steroid injections remains a challenge.

The human body has multiple endogenous mechanisms to regulate and resolve inflammation in response to tissue damage or infection [6]. These include specialized pro-resolving lipid mediators (e.g., resolvins and lipoxins), anti-inflammatory cytokines (e.g., IL-10 and TGF-β), and purinergic signaling pathways [6, 7]. Among these various mechanisms, the adenosine system has gained particular attention for its potent anti-inflammatory and tissue-regenerative capabilities [7]. A line of evidence suggests that purine nucleosides such as adenosine and guanosine can exert strong anti-inflammatory and analgesic effects by activating adenosine receptors (ARs), particularly the A2A subtype, on various immune and joint-resident cells [7]. During inflammatory or degenerative processes, tissue injury causes the extracellular release of ATP, DNA and RNA, and they are subsequently metabolized to adenosine by the ectonucleotidases CD39 and CD73 expressed on activated macrophages [8]. The adenosine thus generated binds to its receptors to increase intracellular cAMP, exerting anti-inflammatory effects and promoting tissue repair [8]. In OA models, adenosine has been shown to modulate the release of pro-inflammatory cytokines, suppress synovial inflammation, and reduce pain signaling [9]. Meanwhile, although guanosine is supported by limited, primarily preclinical data, it also appears to have immunoregulatory effects through A1R and A2AR and may synergize with adenosine through related purinergic pathways [10, 11]. These observations highlight the therapeutic potential of purinergic signaling—particularly the adenosine system—for alleviating inflammation and pain in OA.

Despite its therapeutic potential, adenosine-based therapy faces limitations in clinical application due to its short half-life and the risk of cardiovascular complications. Systemic administration of adenosine results in rapid clearance—its plasma half-life is only a matter of seconds—significantly narrowing the therapeutic window [12]. Moreover, systemic administration of adenosine is known to slow atrioventricular (AV) nodal conduction, potentially causing transient heart block [13]. These challenges underscore the need for a localized, sustained-release drug delivery strategy that maintains effective concentrations of adenosine within the target joint space while minimizing systemic risks. To address these challenges, poly(lactic-co-glycolic) acid (PLGA) nanoparticles have emerged as an attractive drug delivery vehicle [14]. PLGA is biocompatible, biodegradable, and can be formulated to release its cargo over extended periods [14, 15]. By encapsulating nucleoside-based oligonucleotides in PLGA, it becomes possible to protect them from rapid degradation, extend their half-lives, and tailor their release kinetics within the joint [15]. Intra-articular injection of these nanoparticles concentrates the therapeutic agent where it is needed most, potentially enhancing efficacy while minimizing systemic exposure [16]. This approach may overcome the limitations associated with adenosine’s short half-life and cardiac side effects, offering a more targeted and sustained option for OA management [16]. In this study, we explored the therapeutic potential of adenosine- and guanosine-based oligonucleotides delivered via PLGA nanoparticles in an experimental model of OA. We hypothesized that this localized, controlled-release strategy would reduce synovial inflammation, alleviate pain, and ultimately slow disease progression more effectively and safely than traditional treatments. To identify the most potent oligonucleotide, we screened about 500 different 10- to 20-mer sequences for their ability to suppress lipopolysaccharide (LPS)-induced nitric oxide (NO) production. We then encapsulated the most effective candidate within PLGA to achieve targeted, sustained release within the affected joint. The therapeutic efficacy of this PLGA–oligonucleotide complex was examined in an in vivo surgical OA model. We further characterized the mechanism of action by identifying the relevant purinergic receptors and elucidating the molecular events responsible for its anti-inflammatory and analgesic effects. Our findings provide evidence of the purinergic system’s potential as a promising, side-effect-free strategy for mitigating inflammation and pain in OA therapy.

## Materials and methods

### Measurement of nitric oxide

RAW264.7 cells were seeded in 96-well plates at a density of 1 × 10^6^ cells per well. Each well was treated with a unique oligonucleotide from a library of 482 distinct sequences at a final concentration of 10 nM, together with lipopolysaccharide (LPS) at a concentration of 1 µg/mL. The anti-inflammatory effect of each oligonucleotide was evaluated by measuring the reduction in nitric oxide (NO) production compared to LPS-treated group using the nitrate/nitrite colorimetric assay (Cayman Chemical), which quantifies NO concentrations in the culture supernatant.

#### Microfluidics-based PLGA nanoparticles production

The oligonucleotide with the most potent inhibitory effect on NO production in RAW264.7 cells was encapsulated in PLGA nanoparticles using the NanoAssemblr^®^ Ignite instrument (Precision Nanosystems Inc., Canada) with a microfluidics-based approach. The aqueous phase was prepared by dissolving tocopherol polyethylene glycol succinate (TPGS) at 0.3% w/v in pure water, followed by the addition of the selected oligonucleotide at an encapsulation-optimized concentration. The organic phase was prepared by dissolving PLGA at 1% w/v in ethyl acetate. Self-assembly of PLGA nanoparticles was achieved by mixing the organic and aqueous phases in a staggered herringbone chaotic structured microfluidic cartridge (Precision Nanosystems Inc., Canada). The total flow rate was fixed at 12 mL/min, with the aqueous-to-organic flow rate ratio (FRR) maintained at 3:1 by volume. After synthesis, the nanoparticles were filtered through a 0.22 μm syringe filter to remove aggregates and ensure uniform particle size. Subsequently, the nanoparticles were dialyzed against 2 L of DPBS at room temperature for 3 h with gentle stirring at 100 rpm using a dialysis membrane with a molecular weight cut-off (MWCO) of 12–14 kDa (Sigma-Aldrich, St. Louis, MO) to remove residual organic solvent. The purified PLGA nanoparticles were stored at 4 °C until further use.

#### Characterization of PLGA nanoparticles

The synthesized PLGA nanoparticles were characterized using transmission electron microscopy (TEM), nano particle tracking analysis, and zeta-potential analyzer. The morphology and size of the nanoparticles were analyzed using TEM. Silicon wafers were cleaned by sonication in 100% ethanol for 8 min and rinsed with nanopure water. A 20 µL aliquot of the nanoparticle suspension was drop-cast onto the silicon wafers and left to evaporate overnight in a laminar flow hood at room temperature. The samples were imaged using a FEI Magellan 400 microscope equipped with an insertable concentric backscatter detector at a landing energy of 1 kV and a stage bias of 4 kV. Particle size and morphology were analyzed using ImageJ (NIH) software to calculate Feret’s diameter and circularity.

Particle size and concentration were measured using the Nano Particle Tracking Analysis System (Nanosight NS 300, Malvern Panalytical, UK). Nanoparticles were diluted with distilled water to an optimal concentration, and measurements were conducted at room temperature. Video data were analyzed with Nanosight software to determine particle size distribution and particle concentration. The zeta potential of the nanoparticles was measured to assess their surface charge and colloidal stability using a zeta-potential and particle size analyzer (ELSZ-2000ZS, Otsuka Electronics). Samples were diluted in 0.1× PBS to minimize the effects of high ionic strength, and measurements were performed at 25 °C. Each sample was analyzed in triplicate, and the average zeta potential values were recorded.

#### Encapsulation efficacy of PLGA nanoparticle

Encapsulation efficiency (EE) was determined by quantifying non-encapsulated oligonucleotide in the post-centrifugation supernatant. Oligonucleotide-loaded PLGA nanoparticles were dispersed in PBS and centrifuged at 15,700 × g for 50 min at 4 °C to pellet particles. The supernatant was collected without disturbing the pellet, and oligonucleotide concentration was measured on a NanoDrop spectrophotometer (Thermo Fisher Scientific, Waltham, MA) using PBS and a formulation-matched blank for baseline correction. The mass of un-encapsulated oligonucleotide was subtracted from the total input oligonucleotide (1000 µg) to obtain the encapsulated amount and derive the EE.

#### Fourier transform infrared spectroscopy (FT-IR)

Fourier-transform infrared (FT-IR) spectra were acquired using a FT-IR/NIR Spectrophotometer (PerkinElmer, Shelton, CT). Lyophilized nanoparticle samples were measured in the mid-infrared region (4000–400 cm⁻¹) at 4 cm⁻¹ resolution with 32 co-added scans per spectrum (optical path difference velocity 0.5 cm/s). A fresh background was acquired on a clean ATR crystal before each measurement, and the enclosure was purged with dry air when humidity was elevated.

#### Differential scanning calorimetry (DSC) analysis

Thermal transitions were measured on DSC Q2000 instrument (TA Instruments, New Castle, DE) using hermetic Tzero™ aluminum pans. Lyophilized nanoparticle powders (~ 2.0 mg) were crimp-sealed and analyzed with the instrument in Exotherm-Up configuration. Samples were equilibrated at − 50 °C (1 min), heated to 120 °C at 10 °C/min and held 5 min, then cooled to 25 °C at 10 °C/min (1-min hold) before a second heating from 25 °C to 230 °C at 10 °C/min (1-min hold at the plateau). Nitrogen purge gas was supplied at 30 mL/min. Heat flow was recorded continuously and normalized to sample mass.

### Ethics and isolation of primary chondrocytes

This study was conducted in compliance with the guidelines established by the National Research Council (US) Committee for the Care and Use of Laboratory Animals and was approved by the Institutional Review Board of the Kyungpook National University School of Medicine (Daegu, Korea under the approval number KNU 2023 − 0103. All procedures were performed to minimize animal discomfort and in accordance with institutional ethical standards.

Primary chondrocytes were isolated from the articular cartilage of the femurs and tibias of 5-d-old C57BL/6J mice. The harvested cartilage was minced into small fragments and initially incubated with 0.25% trypsin-EDTA at 37 °C for 15 min with gentle agitation to remove connective tissue. Subsequently, the tissue fragments were digested in 0.2% collagenase type II prepared in DMEM at 37 °C for 4 h with continuous gentle shaking to release chondrocytes. After digestion, the resulting cell suspension was filtered through a 40-µm cell strainer to remove undigested debris and washed twice with PBS. The isolated cells were resuspended in complete culture medium consisting of a 3:2 mixture of F12 and DMEM supplemented with 0.25% L-glutamine and 0.25% penicillin/streptomycin. The chondrocytes were seeded in 6-well plates at a density of 1 × 10^5^ cells per well and incubated at 37 °C in a humidified atmosphere with 5% CO2. Only passage 0 chondrocytes were used in the experiments to reduce the dedifferentiation effect of chondrocytes.

#### MTT assay

The effect of the synthesized PLGA nanoparticles on cell viability was assessed using the MTT assay. Primary chondrocytes were seeded in 96-well plates at a density of 1 × 10^5^ cells per well and allowed to adhere overnight. After treatment with IL-1β (10 ng/mL) and varying concentrations of the nanoparticles (0, 2, 20, 200, and 2000 nM) for 24 h, 20 µL of MTT solution (5 mg/mL in PBS) was added to each well and incubated at 37 °C for 4 h. Following incubation, the culture medium was carefully removed, and the formazan crystals formed in viable cells were dissolved by adding 100 µL of dimethyl sulfoxide (DMSO) to each well. The absorbance was measured at 570 nm using a microplate reader, and cell viability was expressed as a percentage relative to untreated control cells.

#### LPS-induced sepsis model

Eight-week-old male C57BL/6J mice were randomly assigned to treatment groups (*n* = 7 per group) and housed under standard conditions with free access to food and water. Endotoxemia was induced by intraperitoneal (IP) injection of LPS at 10 mg/kg in sterile PBS. One hour before LPS administration, mice received a single IP dose of NanoOligo at 10 or 30 mg/kg, free oligonucleotide at 30 mg/kg, dexamethasone at 5 mg/kg, or matched vehicle. Survival was monitored at 4 h intervals for 96 h by observers blinded to group allocation.

#### Surgical OA induction in rat

Male Lister Hooded (Crl: LIS) SPF rats (10 weeks old) were used to induce a surgical OA model. Rats were housed under standard conditions, three per cage, and acclimatized for two weeks prior to surgery. All procedures complied with institutional ethical guidelines for animal care and use. The anterior cruciate ligament transection with partial meniscectomy (ACLT + pMx) was performed under isoflurane anesthesia by transecting the anterior cruciate ligament and excising approximately 50% of the medial meniscus to destabilize the knee joint. Postoperative analgesia was provided with subcutaneous meloxicam (0.5 mg/kg). Rats were randomly divided into groups (seven per group) and received intra-articular injections of PBS, Lorecivivint (0.3 μg in 50 μl), and NanoOligo (10 or 50 µg in 50 μl) on days 7, 21, 35 and 49. Sham-operated controls received PBS injections without ACLT + pMx. Pain and functional impairment were evaluated weekly by measuring the weight bearing index. A dual-channel weight-bearing device was used to record the weight distribution between the affected (right) and unaffected (left) hind limbs. The weight bearing index was calculated as the percentage of body weight supported by the affected limb relative to the total weight borne by both hind limbs, with reduced weight bearing indicating OA-related pain. On day 56 post-surgery, all rats were euthanized under isoflurane anesthesia via transthoracic cardiac puncture. Knee joint tissues were collected for histological and micro-CT imaging analyses.

#### Safranin-O staining and MicroCT imaging

For histological evaluation, harvested knee joints were fixed in 10% formalin for 48 h, and decalcified in 10% ethylenediaminetetraacetic acid (EDTA) solution for 3 weeks at room temperature with regular solution changes. The samples were then embedded in paraffin, sectioned at a thickness of 5 μm, and mounted on slides. The sections were stained with Safranin-O and fast green to assess proteoglycan content in the articular cartilage. Histological images were captured using a light microscope, and the severity of cartilage degradation was graded using the Osteoarthritis Research Society International (OARSI) scoring system which includes stage and total score evaluations [17].

For structural assessment of osteophytes and subchondral bone, knee joints were scanned using a high-resolution micro-computed tomography (microCT) system. Specimens were scanned at a resolution of 9 μm per voxel, with a voltage of 50 kV and current of 200 µA. The acquired images were reconstructed and analyzed using dedicated software to measure osteophyte volume and subchondral bone changes. Quantification of osteophyte volume (mm³) was performed, and representative 3D reconstructions of the knee joints were generated.

#### RNA isolation and quantitative PCR (qPCR)

Primary chondrocytes were cultured and treated with IL-1β (1 ng/mL) and/or NanoOligo (12.5 nM) for the indicated time points. Total RNA was isolated from the cells using TRIzol reagent (Invitrogen, USA) according to the manufacturer’s instructions. RNA concentration and purity were assessed using a NanoDrop spectrophotometer (Thermo Scientific). cDNA was synthesized using a reverse transcription kit (Thermo Fisher Scientific) with 1 µg of total RNA as the template. Quantitative PCR (qPCR) was performed using SYBR Green PCR Master Mix (Applied Biosystems) on a StepOnePlus Real-Time PCR System (Applied Biosystems). The expression levels of Mmp3, Mmp13, Adamts5, Tnf-α, Il-6, Vegf, and ARs were measured, with GAPDH used as the internal control for normalization. Relative mRNA expression levels were calculated using the ΔΔCt method and expressed as fold changes compared to the control group. Primer sequences for each gene were designed using Primer-BLAST and verified for specificity.

#### Western blot analysis

Primary chondrocytes were lysed in RIPA buffer containing a protease and phosphatase inhibitor cocktail (Thermo Fisher Scientific). Protein concentration was determined using a bicinchoninic acid (BCA) protein assay kit (Pierce). Equal amounts of protein (20 µg) were separated on 10–12% SDS-PAGE gels and transferred onto polyvinylidene fluoride (PVDF) membranes (Millipore). Membranes were blocked in 5% non-fat dry milk prepared in Tris-buffered saline with 0.1% Tween-20 (TBST) for 1 h at room temperature. The membranes were incubated overnight at 4 °C with primary antibodies specific to MMP3 (Abcam, Cambridge, UK, #ab52915), MMP13 (#ab51072), ADAMTS-5 (#ab41037), IL-1β (Cell Signaling Technology, Beverly, MA, #CS2022), TNF-α (#CS3707), IL-6 (#ab6672), IL-10 (#ab33471), p-PKA (#CS5661), PKA (#CS4782), p-CREB (#CS9198), CREB (#CS9197), p-AMPKα (#CS2531), AMPKα (#CS2532), p-p38 (#CS4631), p38 (#CS9212), p-ERK1/2 (#CS9101), ERK1/2 (BD #610123), p-IkB (#CS9246), IkB (#CS4814), p-p65 (#CS3033), p65 (#CS8242), p-FoxO3 (#CS9466), FoxO3a (#CS2497), Sirt1 (#CS9475), Nrf2 (#CS12721), HO-1 (#CS70081) and β-actin (Sigma-Aldrich, Burlington, MA, #A1978). After washing with TBST, membranes were incubated with horseradish peroxidase (HRP)-conjugated secondary antibodies for 1 h at room temperature. Protein bands were detected using an enhanced chemiluminescence (ECL) detection kit (Amersham) and visualized with an imaging system (Bio-Rad). Quantitative densitometric analysis was performed using ImageJ software, and protein expression was normalized to β-actin. Western blot experiments were performed in biological triplicates (three independent experiments) to ensure reproducibility. Quantified protein expression levels normalized to internal control were represented as mean ± SD in the corresponding graphs.

#### Reactive oxygen species (ROS) detection using DHE and mitosox staining

Primary chondrocytes were seeded on coverslips in 24-well plates and treated with IL-1β (1 ng/mL) in the presence or absence of NanoOligo (6.25, 12.5, or 25 nM) or Lorecivivint (10 nM) as a positive control. After 24 h of treatment, cells were incubated with dihydroethidium (DHE; 5 µM, Thermo Fisher Scientific) or MitoSOX Red (5 µM, Thermo Fisher Scientific) for 30 min at 37 °C in the dark. For DHE staining, fluorescence images were captured using a fluorescence microscope, and the percentage of DHE-positive cells was quantified. For MitoSOX staining, cells were washed with PBS after incubation, and fluorescence images were captured to determine the percentage of MitoSOX-positive cells.

#### Immunofluorescent 8-oxo-dG staining

To detect oxidative DNA damage, cells were fixed with 4% paraformaldehyde for 15 min, permeabilized with 0.1% Triton X-100, and incubated with an anti-8-oxo-dG antibody (1:200, Santa Cruz Biotechnology, #sc-66036) overnight at 4 °C. Cells were then incubated with a fluorophore-conjugated secondary antibody (1:500, Jackson ImmunoResearch Laboratories, West Grove, PA) for 1 h at room temperature. Fluorescence was visualized using a fluorescence microscope, and the percentage of 8-oxo-dG-positive cells was calculated.

#### Senescence detection using β-galactosidase staining

To evaluate cellular senescence, β-galactosidase staining was performed using a senescence-associated β-galactosidase staining kit (Cell Signaling Technology). After treatment with IL-1β (1 ng/mL) in the presence or absence of NanoOligo or Lorecivivint for 48 h, cells were washed with PBS and fixed with 4% paraformaldehyde for 15 min at room temperature. The cells were then incubated with β-galactosidase staining solution at 37 °C for 12–16 h in a CO₂-free environment. Stained cells were imaged under a bright-field microscope, and the β-gal-positive area was quantified using ImageJ software.

### Statistical analysis

All data were presented as mean ± standard deviation (SD). Two-group comparisons were performed with the Mann–Whitney U test. For comparisons involving multiple groups, post hoc *p*-values were adjusted for multiplicity using the Benjamini–Hochberg false discovery rate (FDR) procedure. Survival was analyzed by the Kaplan–Meier method, and group curves were compared using two-sided log-rank tests. Two-sided *P* ≤ 0.05 was considered statistically significant. Analyses were performed in R (version 4.5.2.; R Foundation for Statistical Computing) and GraphPad Prism (version 8; GraphPad Software, San Diego, CA).

## Results

### Identification and characterization of a potent anti-inflammatory oligonucleotide encapsulated in PLGA nanoparticles

**Fig. 1:**
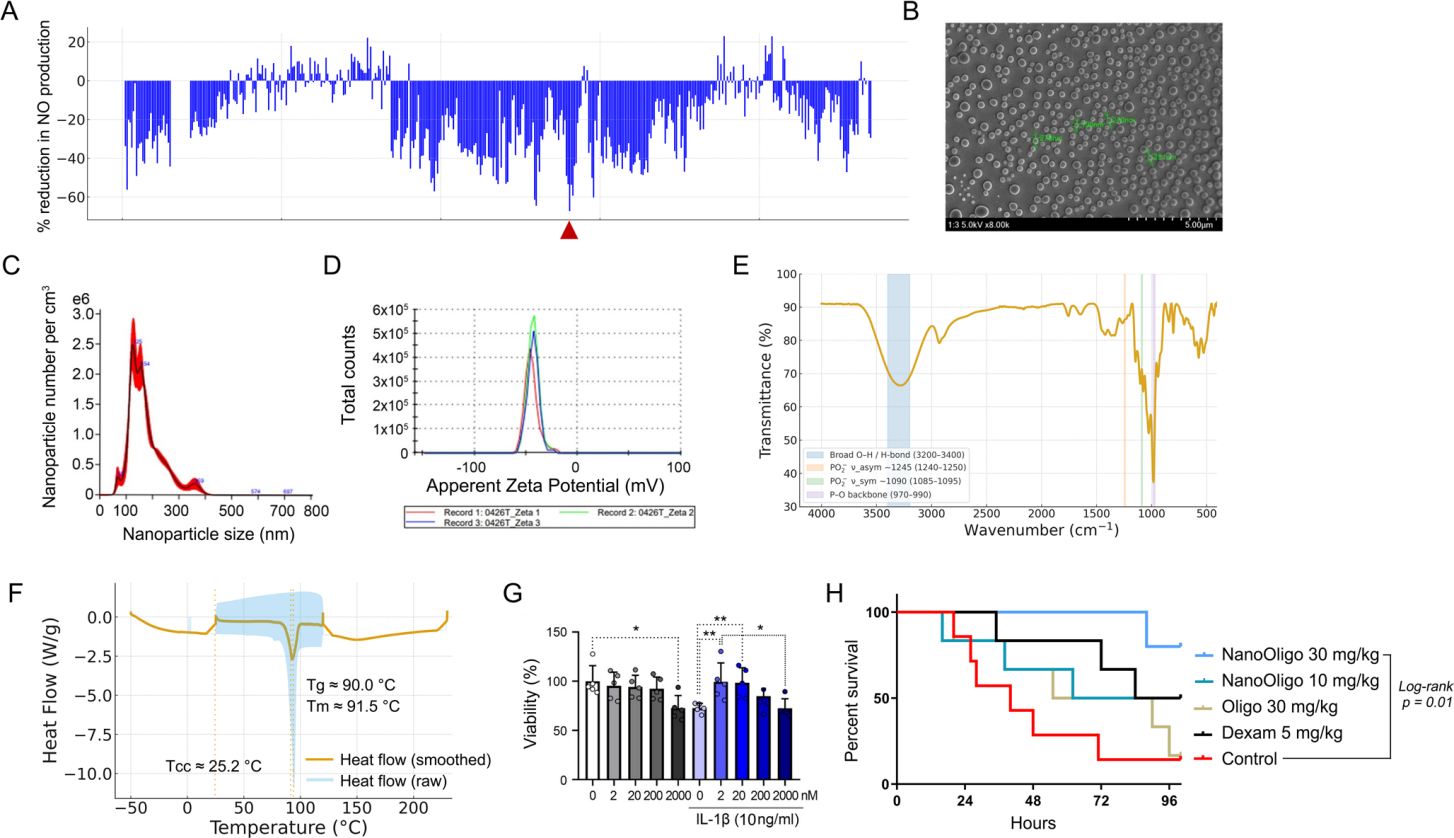
Screening of anti-inflammatory oligonucleotides and characterization of PLGA nanoparticle-encapsulated oligonucleotides (NanoOligo). (**A**) To find the oligonucleotide sequence with the most potent anti-inflammatory effect, RAW 264.7 cells were treated individually with 482 oligonucleotides at 10 nM alongside LPS (1 µg/mL), and NO production was quantified by a colorimetric assay performed in triplicate (*n* = 3). The red triangles on the x-axis indicate the oligonucleotide that most reduced NO production. (**B**) Transmission electron microscopy image of PLGA nanoparticles encapsulating the oligonucleotide. The sizes of indicated PLGA nanoparticles were measured and indicated. (**C**) Particle size distribution of the PLGA nanoparticles was measured using Nanoparticle tracking analysis. The average size of LNPs was 121 nm. (**D**) Zeta potential of the PLGA nanoparticles was analyzed and the average was − 42 mV. (**E**) FT-IR analysis of the oligonucleotide–PLGA formulation. FT-IR spectrum (Transmittance, %) with key oligonucleotide bands highlighted; a broad O–H/H-bond stretch at 3200–3400 cm⁻¹ (blue band), PO₂⁻ asymmetric stretch ~ 1245 cm⁻¹ (1240–1250; orange line), PO₂⁻ symmetric stretch ~ 1090 cm⁻¹ (1085–1095; green line), and P–O backbone ~ 970–990 cm⁻¹ (purple band). (**F**) Differential scanning calorimetry (DSC) thermogram. The light-blue shaded area indicates the raw heat-flow trace, and the orange line shows the smoothed trace. The endothermic direction is downward. Labeled features include the cold crystallization (Tcc = 25.2 °C), glass transition (Tg = 90.0 °C), and melting peak (Tm = 91.5 °C). (**G**) The effect of PLGA nanoparticles on cell viability was assessed using an MTT assay following treatment with various concentrations of LNPs in the presence of IL-1β (10 ng/ml). *p < 0.05, **p < 0.01. Mann–Whitney U test. (**H**) Survival in an LPS-induced sepsis model. Kaplan–Meier survival curves for mice challenged intraperitoneally with lipopolysaccharide 10 mg/kg and treated with PBS control, dexamethasone 5 mg/kg, Oligo 30 mg/kg, NanoOligo 10 mg/kg, or NanoOligo 30 mg/kg. Log-rank test, *n* = 7 per group

To identify the most potent anti-inflammatory oligonucleotide sequence, 482 distinct oligonucleotides were each administered at a final concentration of 10 nM to RAW 264.7 macrophage cell-lines in the presence of 1 µg/mL LPS, and nitric oxide (NO) production was quantified. Several candidate oligonucleotides markedly suppressed LPS-induced NO release, and the top-performing sequences were selected for subsequent nanoparticle encapsulation studies. After further validation, the following sequence was ultimately chosen for the experiment: AGG-GAG-GGA-GGG-AGG-GAT (Fig. 1A). The top 30 and bottom 30 oligonucleotides for suppression of LPS-induced NO production have been added to Supplementary Tables 3 and 4.

PLGA nanoparticles encapsulating the leading oligonucleotide candidate were characterized for morphology, size distribution, surface charge, and encapsulation properties. TEM revealed that the nanoparticles had a generally spherical shape with diameters ranging from approximately 100 to 300 nm (Fig. 1B). This result was confirmed by nanoparticle tracking analysis (NTA), which showed a primary size peak at approximately 121 nm (Fig. 1C). Zeta potential measurements yielded a mean value of − 42 mV (Fig. 1D), confirming the nanoparticles had a negatively charged surface. Encapsulation efficiency of oligonucleotide into PLGA nanoparticle across replicates was 36.96% ± 3.76% (Supplementary Table 5). Fourier transform infrared spectroscopy (FT-IR) spectra revealed the concurrent signatures from of PLGA and the oligonucleotide: the PLGA ester C = O band at ~1754 cm⁻¹ and the phosphate-backbone bands of the oligonucleotide at ~1245, ~1090, and 970–990 cm⁻¹ were all present. The broad O–H/N–H stretching region (3200–3400 cm⁻¹) was enhanced, while no new absorption bands appeared across 4000–500 cm⁻¹, supporting physical encapsulation rather than chemical conjugation between PLGA and the oligonucleotide (Fig. 1E). Differential scanning calorimetry (DSC) revealed a single glass-transition near 90.0 °C and an endothermic event centered at ~ 91.5 °C, with a cold-crystallization feature around 25.2 °C; no additional thermal events were detected within the scanned range (Fig. 1F). Taken together, the single dominant transition set in DSC, alongside an unchanged FT-IR peak pattern, is consistent with the oligonucleotide being appropriately encapsulated within the PLGA matrix rather than chemically bound to it.

To assess biocompatibility and potential cytotoxicity under inflammatory conditions, RAW 264.7 cells were treated with various concentrations of PLGA-encapsulated oligonucleotides (NanoOligo) in the presence of 10 ng/mL IL-1β and subjected to MTT assays. NanoOligo exhibited no significant cytotoxicity at concentrations below 200 nM, while viability declined markedly at 2,000 nM. Under IL-1β-induced inflammatory stress, 2 nM and 20 nM NanoOligo restored viability, whereas the 2,000 nM dose caused a statistically significant reduction relative to the 2 nM group (Fig. 1G).

To determine whether PLGA nanoparticle encapsulation enhances anti-inflammatory efficacy over free oligonucleotide, mice were challenged intraperitoneally with LPS (10 mg/kg) and monitored for 96 h. Free oligonucleotide (30 mg/kg) produced an early survival benefit that was lost by 96 h, becoming indistinguishable from control. By contrast, NanoOligo 10 and 30 mg/kg yielded survival curves comparable to the positive control dexamethasone (5 mg/kg), and only NanoOligo 30 mg/kg achieved a statistically significant improvement versus control (log-rank *p* = 0.01), supporting superior efficacy of the nanoparticle formulation over free oligonucleotide in the sepsis model.

### Therapeutic efficacy of NanoOligo in an ACLT-pMx-induced rat model of OA

**Fig. 2:**
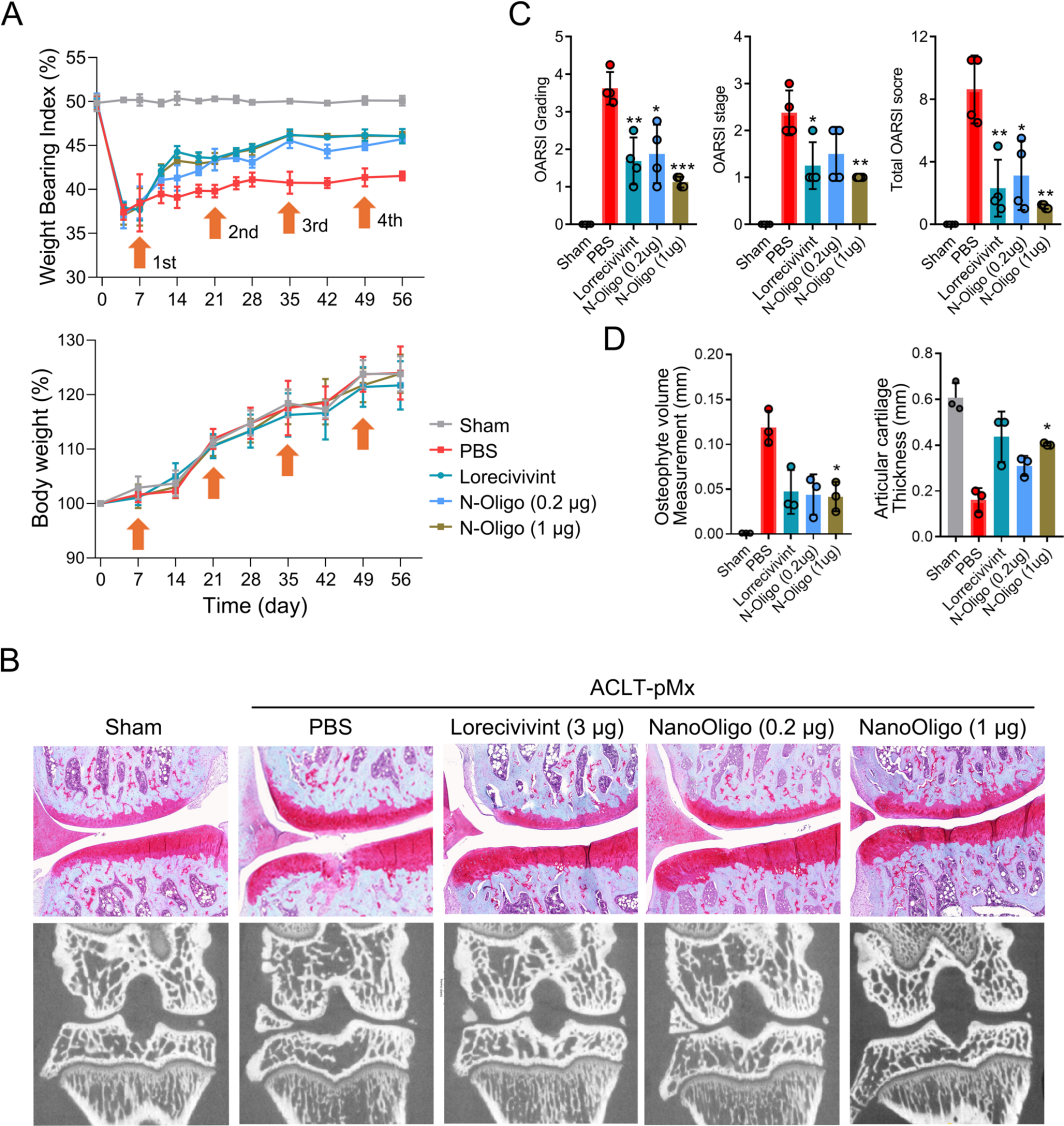
Assessment of therapeutic efficacy and toxicity of NanoOligo in an ACLT-pMx-induced rat OA model. (**A**) OA was induced in 10-week-old rats using ACLT-pMx surgery. Pain was assessed by measuring the weight-bearing index, and body weight was monitored weekly to evaluate potential drug toxicity. NanoOligo (10 or 50 µg in 50 μl) and Lorecivivint (0.3 μg in 50 μl), as a positive control for therapeutic efficacy, were administered via intra-articular injections on days 7, 21, 35, and 49 post-surgery. *N* = 7 per group. (**B**) Articular cartilage degeneration was evaluated by Safranin-O staining (*N* = 4 per group), and structural changes, including osteophyte formation and subchondral bone integrity, were analyzed by microCT imaging (*N* = 3 per group). (**C**) Histological analysis included OARSI grading, staging, and total OARSI score quantification to assess the severity of cartilage damage. (**D**) MicroCT analysis quantified osteophyte volume and articular cartilage thickness. *p < 0.05, **p < 0.01, ***p < 0.001 compared to PBS-treated control rat. Mann–Whitney U test

To evaluate the therapeutic efficacy of NanoOligo in surgically induced OA model, rats underwent ACLT-pMx surgery and were treated with intra-articular injections of different doses of NanoOligo (10 or 50 µg in 50 μl) or Lorecivivint as a positive control on days 7, 21, 35, and 49 post-surgery. Functional impact and safety were assessed by monitoring the weight-bearing index and body weight weekly. Improvements in the weight-bearing index were evident in NanoOligo-treated groups, similar to those in Lorecivivint group, suggesting analgesic and functional benefits. Furthermore, NanoOligo treatment did not significantly affect body weight, indicating an acceptable safety profile (Fig. 2A). Safranin-O staining revealed more enhanced proteoglycan retention and reduced cartilage erosion in the NanoOligo-treated groups relative to PBS-treated controls (Fig. 2B, upper panels). Correspondingly, microCT images demonstrated reduced osteophyte formation and improved subchondral bone integrity in NanoOligo-treated rats compared to PBS controls (Fig. 2B, lower panels). Quantitative histological assessments confirmed these observations, with significant reductions in OARSI grading, staging, and total scores, indicating robust cartilage protection by NanoOligo (Fig. 2C). Additionally, microCT-based analysis showed a marked decrease in osteophyte volume and modest improvements in articular cartilage thickness in 50 µg NanoOligo-treated group (Fig. 2D). To further confirm these findings, we evaluated the therapeutic potential of NanoOligo in a mouse destabilization of the medial meniscus (DMM) model, administering intra-articular injections at 1, 3, 5 and 7 week post-surgery. Consistent with our rat OA findings, NanoOligo-treated mice showed significantly lower total OARSI scores, reduced subchondral bone thickness, and smaller osteophyte sizes compared to controls (Supplementary Fig. 1A, B).

### Adenosine receptor-dependent anti-catabolic effects of NanoOligo in primary chondrocytes

**Fig. 3:**
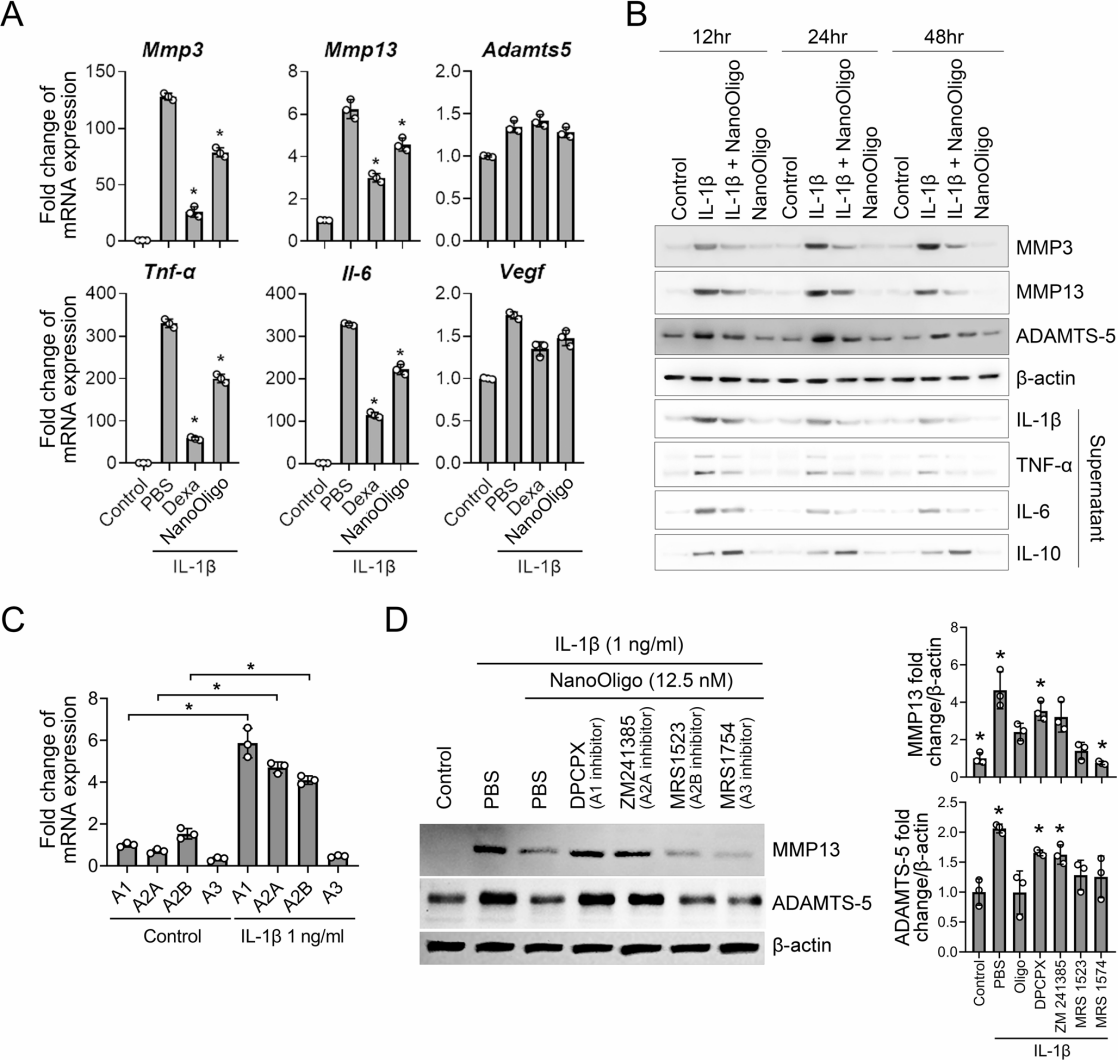
Anti-catabolic effects of NanoOligo are dependent on A1R and A2AR in primary chondrocytes. (**A**) Primary chondrocytes were treated with PBS, dexamethasone (1 µM), or NanoOligo (12.5 nM) in the presence of IL-1β (1 ng/mL) for 6 h, and the gene expression of *MMP3*,* MMP13*,* Adamts5*,* Tnfα*,* IL-6*, and *Vegf* was assessed by qPCR. *p < 0.05 compared to PBS control by Mann–Whitney U test. (**B**) Primary chondrocytes were treated with or without NanoOligo (12.5 nM) in the presence of IL-1β for 12, 24, and 48 h. Protein expression of MMP3, MMP13, and Adamts5 was evaluated in total cell lysate, and cytokine levels of IL-1β, Tnfα, IL-6, and IL-10 were also measured in the supernatant. (**C**) The gene expression of ARs in primary chondrocytes was assessed after 6 h of treatment with or without IL-1β. *p < 0.05 by Mann–Whitney U test. (**D**) To investigate whether the anabolic effect of NanoOligo is mediated through ARs, primary chondrocytes were treated with AR inhibitors together with NanoOligo. The changes in MMP13 expression induced by IL-1β were quantified and represented in the graph. *p < 0.05 compared to NanoOligo treated group by Mann–Whitney U test

To investigate whether NanoOligo exerts anti-catabolic effects in primary chondrocytes, we first quantified the expression of matrix-degrading enzymes and inflammatory mediators following IL-1β treatment. Co-treatment with NanoOligo (12.5 nM) significantly suppressed the IL-1β-induced upregulation of *Mmp3*,* Mmp13*,* Tnfα*, and *IL-6*, although this effect was less pronounced than that of dexamethasone. NanoOligo did not affect the transcription of *Adamts5* or *Vegf* (Fig. 3A). To confirm these observations at the protein level, we then evaluated MMP3, MMP13, and ADAMTS-5 expression at 12, 24, and 48 h. Consistent with the gene expression data, NanoOligo markedly reduced MMP3 and MMP13 production. Notably, ADAMTS-5 protein levels were also diminished, even though its transcript level remained relatively unchanged. Furthermore, the secretion of pro-inflammatory cytokines such as IL-1β, TNFα, and IL-6 was substantially decreased, while IL-10 was increased, suggesting that NanoOligo shifts the chondrocyte environment toward anti-inflammatory state (Fig. 3B).

Given the established role of ARs in purinergic signaling pathway, we then investigated whether the anti-catabolic effects of NanoOligo were mediated through these receptors. Under IL-1β-induced catabolic conditions, primary chondrocytes showed significantly increased expression of A1R, A2AR, and A2BR (Fig. 3C). Pharmacological inhibition of A1R with DPCPX or A2AR with ZM241385 largely abrogated the NanoOligo-induced suppression of MMP13 and ADAMTS-5, indicating that NanoOligo exerts its robust anti-catabolic effects via pathways involving A1R and A2AR. In addition, inhibition of A3R with MRS1754 further decreased MMP13 protein levels, suggesting that A3R-related signaling is involved in MMP13 production (Fig. 3D).

### NanoOligo activates PKA–CREB and leads to a rapid attenuation in p38 MAPK signaling under IL-1β-induced catabolic condition

**Fig. 4:**
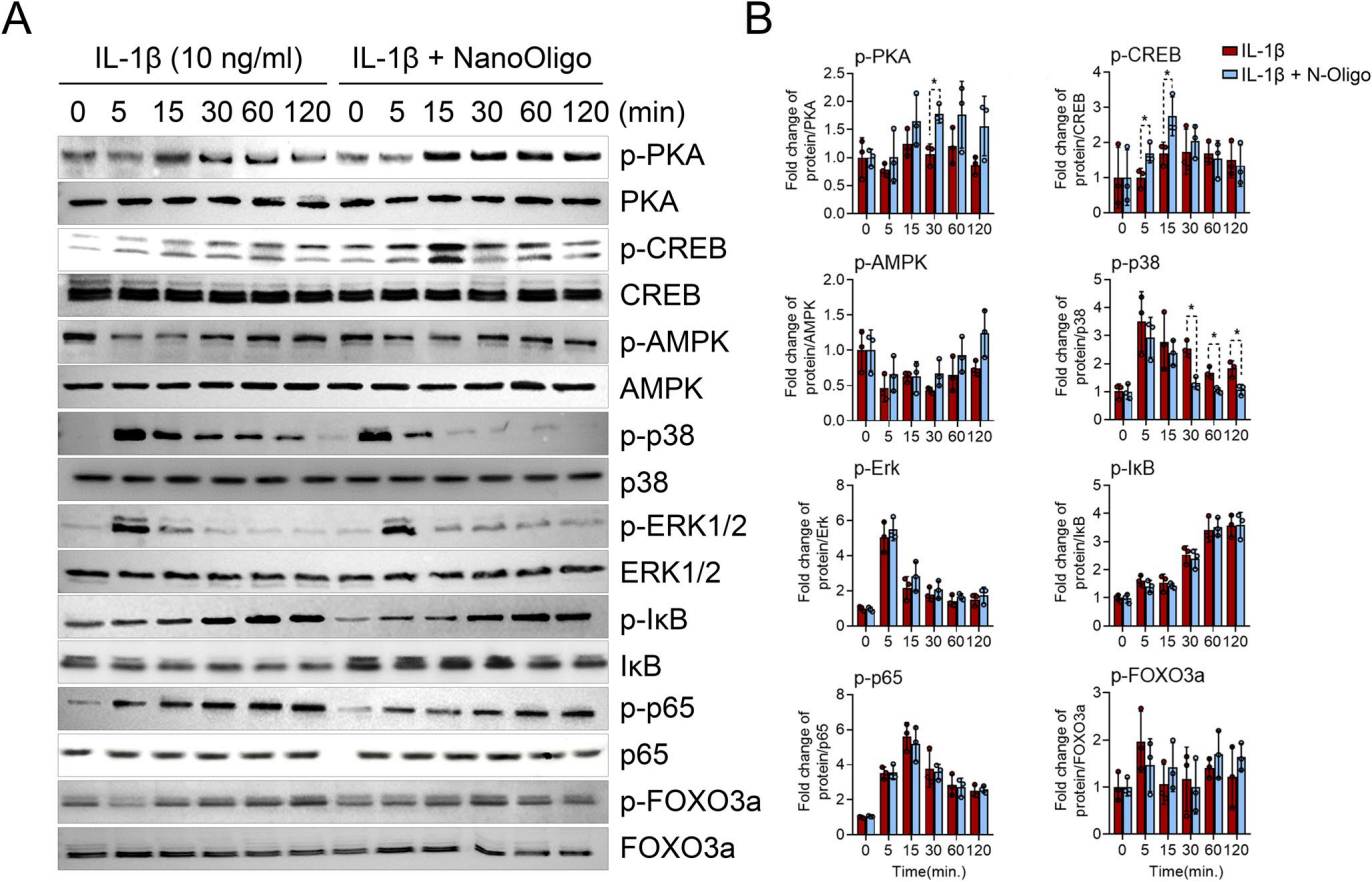
NanoOligo activates the PKA-CREB signaling pathway and rapidly decreases p38 MAPK activation under IL-1β–induced catabolic conditions. (**A**) Primary chondrocytes were serum-starved overnight and then stimulated with IL-1β (10 ng/mL), with or without NanoOligo (12.5 nM). Whole-cell lysates were harvested at the indicated time points and subjected to Western blot analysis to detect total and phosphorylated forms of PKA, CREB, AMPK, p38, ERK1/2, IκB, p65, and FOXO3a. (**B**) Densitometric analysis of phosphorylated protein levels, normalized to the corresponding total protein, was performed using ImageJ. Data are presented as fold changes relative to the 0 min control. *p < 0.05 compared to IL-1β-treated group by Mann–Whitney U test (*n* = 3)

We then assessed how NanoOligo influences the time-course activation of cAMP-related and catabolic signaling in primary chondrocytes exposed to IL-1β–treated conditions. Treatment with NanoOligo led to a significant increase in p-PKA and p-CREB levels, suggesting enhanced activation of the PKA–CREB axis, which is generally associated with anabolic responses. In contrast, phosphorylation of p38 MAPK was similarly activated at 5-minute following IL-1β treatment, but thereafter, it showed a more rapid decline in the NanoOligo-treated group compared to the control group. However, the phosphorylation levels of ERK, AMPK, IκB, p65, and FOXO3a were not substantially altered by NanoOligo (Fig. 4). Collectively, the anabolic effects of NanoOligo can be explained by modulation of PKA–CREB pathway and an enhanced decline of p38 signaling.

### NanoOligo mitigates IL-1β–induced oxidative stress and senescence in primary chondrocytes via the Sirt1/Nrf2 axis and HO-1 antioxidant system

**Fig. 5:**
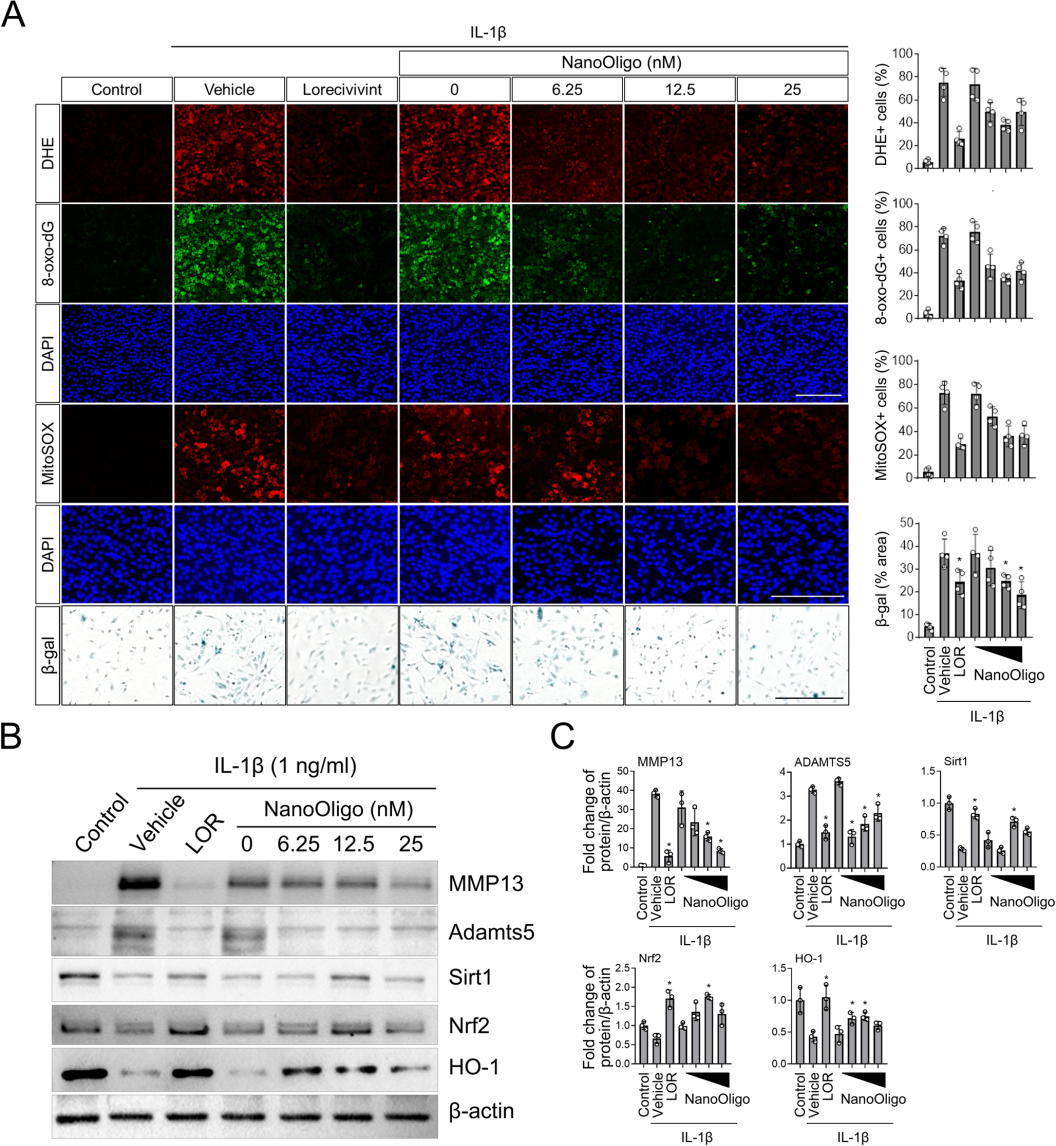
NanoOligo mitigates IL-1β–induced oxidative stress and senescence via Sirt1, Nrf2, and HO-1. (**A**) Primary chondrocytes were treated with IL-1β (1 ng/mL) in the presence of NanoOligo or Lorecivivint (10 nM) at the indicated concentrations for 24 h. Intracellular ROS, oxidative DNA damage, and mitochondrial ROS levels were monitored using DHE, 8-oxo-dG, and MitoSOX staining, respectively, at 24 h, while cellular senescence was assessed by β-galactosidase staining at 48 h. (**B**) After 24 h of IL-1β treatment with or without NanoOligo or Lorecivivint, total protein lysates were harvested and subjected to Western blot analyses of MMP13, ADAMTS5, Sirt1, Nrf2, and HO-1. (**C**) Densitometric quantification of the Western blots was performed using ImageJ, normalized to β-actin, and presented as fold changes relative to untreated control cells. *p < 0.05 compared to IL-1β-treated vehicle group by Mann–Whitney U test

To further elucidate the mechanism underlying chondroprotective effects of NanoOligo, we investigated changes in ROS production and cellular senescence under IL-1β-induced catabolic conditions. Primary chondrocytes treated with IL-1β exhibited significant increases in total ROS, mitochondrial ROS, and oxidative DNA damage, as determined by DHE, MitoSOX, and 8-oxo-dG staining, respectively. Notably, NanoOligo treatment significantly reduced the proportions of DHE+, MitoSOX+, and 8-oxo-dG + cells in a dose-dependent manner compared to the IL-1β-treated vehicle group, with reductions comparable to those observed with Lorecivivint as a positive control. Furthermore, β-galactosidase staining revealed that NanoOligo at 12.5 and 25 nM significantly diminished the accumulation of senescent chondrocytes under these conditions (Fig. 5A).

Based on the reduction in oxidative stress, we analysed the molecular mechanisms regulating the endogenous oxidative stress control system. NanoOligo treatment not only dose-dependently reduced levels of MMP13 and ADAMTS5 but also markedly enhanced the expression of critical antioxidant regulators, such as Sirt1, Nrf2, and HO-1. Notably, the increases in band intensity of Sirt1, Nrf2, and HO-1 were most evident at 12.5 nM rather than at the higher dose of 25 nM, achieving levels comparable to those observed with Lorecivivint (Fig. 5B, C).

## Discussion

Accumulating evidence supports that the endogenous purinergic system modulates inflammation and facilitates tissue repair through the activation of ARs [7, 9]. In this study, we evaluated the therapeutic potential of adenosine- and guanosine-based oligonucleotides encapsulated in PLGA nanoparticles to reduce pain, and slow OA progression in surgically induced OA model. Screening 482 oligonucleotides identified a potent candidate, which was encapsulated in PLGA nanoparticles. Intra-articular injections of NanoOligo in a surgery-induced OA rat model preserved cartilage integrity and improved weight bearing compared to controls. Mechanistically, NanoOligo activated A1R and A2AR, stimulated the PKA–CREB signaling pathway, and inhibited the p38 MAPK pathway. Moreover, NanoOligo reduced oxidative stress and cellular senescence through activation of the Sirt1–Nrf2–HO-1 antioxidant system.

Adenosine and guanosine, the primary components of NanoOligo, exhibit distinct anti-inflammatory profiles characterized by differences in receptor affinity, degradation kinetics, and signaling mechanisms. Adenosine strongly interacts with A1R, A2AR, A2BR, and A3R, particularly elevating intracellular cAMP levels through the A2AR and A2BR, leading to significant anti-inflammatory effects [18, 19]. However, its rapid enzymatic degradation by adenosine deaminase (ADA) limits its therapeutic duration [12]. In contrast, guanosine can potentiate adenosine-induced cAMP signaling [20]. Recent study revealed that guanosine’s neuroprotective actions depend on A2AR expression but do not involve direct binding to A1R, A2AR, or the A1R–A2AR heteromer. Instead, guanosine allosterically modulates A2AR signaling in cells co-expressing A1R–A2AR [21]. This heteromer-dependent modulation allows guanosine to involve A2AR-driven responses, including cAMP accumulation and its downstream signaling pathways, without acting as a conventional agonist or antagonist [21]. Moreover, guanosine is resistant to ADA-mediated degradation, affording it a longer biological half-life compared to adenosine [22]. Beyond receptor heteromer modulation, guanosine also exerts anti-inflammatory actions through alternative pathways involving glutamate receptors and potassium channels, which contribute to reduced oxidative stress and suppression of pro-inflammatory cytokine production [11, 23–25]. Taken together, the protective activity of guanosine stems from both AR-dependent and AR-independent processes, affording it durable anti-inflammatory benefits. Hence, combining adenosine and guanosine in NanoOligo harnesses both the rapid effects of AR signaling and the longer-lasting AR-independent benefits, making NanoOligo a compelling candidate for the management of inflammatory and degenerative conditions such as OA.

Our data revealed that inhibiting A1R and A2AR with its antagonist partially restored NanoOligo-mediated suppression of MMP13 and Adamts5, confirming that NanoOligo acts through both A1R and A2AR (Fig. 4D). ARs were initially classified as either A1 or A2 based on whether they decrease or increase cAMP, respectively [26]. While the anti-inflammatory function of the A2AR is well-established, the contribution of the A1R to NanoOligo’s anti-inflammatory activity appears less convincing. Typically, activation of the A1R lowers cAMP levels through Gi-mediated inhibition of adenylyl cyclase, subsequently activating catabolic kinase pathways such as PKC, PI3 kinase, and MAP kinases [27]. However, our findings suggest that NanoOligo may serve as a partial agonist at A1R, thereby shifting A1R signaling from a predominantly catabolic to a more anabolic or protective role. This is consistent with earlier work showing that A1R partial agonists can modestly increase cAMP—likely through low-affinity Gs engagement—while still producing significant anti-inflammatory outcomes [28]. Such partial agonists, while weaker than full agonists, can nonetheless elevate cAMP enough to support anti-inflammatory outcomes. Furthermore, A1R itself appears to exert additional cartilage-protective effects independent of cAMP modulation, as A1R deletion in ADA-deficient mice heightened inflammation and tissue damage, with increases in Th2 cytokines, chemokines, and matrix metalloproteinases [29]. Collectively, these results indicate that NanoOligo’s anti-inflammatory effects arise from coordinated modulation of both A1R and A2AR, promoting balanced inflammatory signaling and tissue homeostasis.

We found in Fig. 3A and B that *ADAMTS5* remained unchanged after IL-1β stimulation and NanoOligo treatment, whereas ADAMTS5 protein was reduced by NanoOligo. This transcript–protein discordance aligns with prior evidence that ADAMTS5 is the predominant, constitutively expressed aggrecanase in OA cartilage in vivo, while IL-1β does not induce *ADAMTS5* mRNA in standard monolayer cultures [30, 31]. Under these in vitro conditions, monolayer-induced partial dedifferentiation of chondrocytes likely reshapes stimulus–response properties and contributes to transcript–protein uncoupling, although the precise circuitry remains unresolved. Within this context, the most parsimonious interpretation is post-transcriptional control of ADAMTS5 abundance—namely, accelerated proteasomal/lysosomal turnover—yielding lower protein despite stable transcripts. In addition, a non–mutually exclusive mechanism is miRNA-mediated translational repression with minimal mRNA decay, a recognized regulatory mode in mammalian cells, including chondrocytes [32, 33]. Finally, temporal dynamics can accentuate mismatch. The transient mRNA fluctuations may be translated earlier, with transcripts returning toward baseline by our sampling time while protein remains depressed—a pattern widely reported in RNA–protein time-course studies [34].

NanoOligo robustly activated the PKA–CREB pathway under IL-1β-stimulated conditions, consistent with its proposed mechanism of action via ARs (Fig. 4). Notably, NanoOligo treatment suppressed the persistence of p38 MAPK signaling, despite previous reports indicating direct phosphorylation of p38 MAPK by PKA [35]. A closer look at the signaling kinetics revealed p38 MAPK phosphorylation levels were similar in control and NanoOligo-treated cells at 5 min post-IL-1β stimulation; however, a substantial reduction was evident at later time points in NanoOligo-treated chondrocytes (Fig. 4). This finding suggests that NanoOligo does not directly inhibit the initial IL-1β-driven activation of p38 MAPK but rather rapidly terminates its activity, likely through indirect mechanisms involving reduced ROS generation. NanoOligo-mediated activation of the PKA–CREB pathway may enhance antioxidant responses via the Sirt1–Nrf2–HO-1 axis, ultimately attenuating ROS-dependent p38 MAPK signaling [36, 37]. Collectively, these findings emphasize the intricate interplay between anabolic signaling and redox homeostasis, offering valuable insights into potential therapeutic approaches for inflammation and cartilage degeneration in OA.

Interestingly, while NanoOligo treatment reduced MMP13 protein levels in a dose-dependent manner, Nrf2, HO-1, and SIRT1 showed maximal induction at 12.5 nM NanoOligo, whereas the response was attenuated at 25 nM (Fig. 5). Several mechanisms could account for this non-linear pattern. From a pharmacological perspective, adenosine receptor subtype cross-activation may play a role, where low-to-intermediate concentrations activate A2A and A2B receptors to stimulate the cAMP/CREB–Nrf2–HO-1/SIRT1 axis, while higher concentrations additionally engage A1 and A3 receptors with Gi coupling that counterbalance these signals [38]. In addition, receptor desensitization and internalization upon sustained high-level stimulation may also contribute to the decline in downstream responses [39]. Beyond receptor pharmacology, degradation and release dynamics of NanoOligo may be optimal at mid-range concentrations, leading to efficient generation of nucleosides such as adenosine in the extracellular space, whereas higher concentrations may promote nanoparticle aggregation, excessive protein corona formation, or nuclease saturation, thereby reducing the effective availability of active degradation products to stimulate receptor signaling [40, 41]. Finally, activation of innate immune sensors such as toll-like receptor 9, protein kinase R, or cyclic GMP-AMP synthase (cGAS)–stimulator of interferon genes (STING) at high oligonucleotide concentrations may trigger integrated stress responses, suppressing global translation and attenuating Nrf2, HO-1, and SIRT1 expression [42]. Collectively, these findings suggest that the concentration-dependent biphasic response arises from a complex interplay between receptor pharmacology, extracellular degradation kinetics, and stress-response feedback mechanisms.

## Conclusion

In conclusion, adenosine- and guanosine-based oligonucleotides exert their anti-inflammatory effects by activating A1R and A2AR. This activation not only inhibits catabolic signaling via the PKA–CREB pathway but also modulates oxidative stress through the Sirt1–Nrf2–HO-1 system, which may contribute to the attenuation of OA-related catabolic progression. Our data suggests the potential of the endogenous purinergic system as a novel therapeutic strategy for OA.

## Supplementary Information

Below is the link to the electronic supplementary material.

Supplementary Material 1

## Data Availability

All data generated or analyzed during this study are included in this published article and its supplementary data files.
