# Supplementary material for: Adenosine and guanosine-based oligonucleotides-loaded PLGA nanoparticles attenuates progression of surgically induced osteoarthritis
Source: Drug Deliv Transl Res. 2025 Dec 15;16(8):2773–87. doi: 10.1007/s13346-025-02020-6 (PMC13346226; doi:10.1007/s13346-025-02020-6)

**Adenosine and guanosine-based oligonucleotides-loaded PLGA nanoparticles attenuates progression of surgically induced osteoarthritis**

Yoonhee Kim^1,2^, Jin Han^1,2,3^, Ji Young Park^1,4^ and Seungwoo Han^1,2^

**SUPPLEMENTARY TABLE**

Supplementary Table1. Primer List used for qPCR Analysis in this study. The table includes the gene name, primer sequences for both forward and reverse primers, and the corresponding amplicon size.

| Gene | Primer | Primer sequences |
| --- | --- | --- |
| Mmp3 | forward | ACA TGG AGA CTT TGT CCC TTT TG |
|  | reverse | TTG GCT GAG TGG TAG AGT CCC |
| Mmp13 | forward | TGT TTG CAG AGC ACT ACT TGA A |
|  | reverse | CAG TCA CCT CTA AGC CAA AGA AA |
| Adamts5 | forward | CCA TCT TCC CGG TTG TGT ATC T |
|  | reverse | ACT GTT TCA CTC TGG GCT CTT |
| Tnfα | forward | ACG GCA TGG ATC TCA AAG AC |
|  | reverse | GTG GGT GAG GAG CAC GTA GT |
| IL-6 | forward | CCG GAG AGG AGA CTT CAC AG |
|  | reverse | TCC ACG ATT TCC CAG AGA AC |
| Vegf | forward | GAG AGA GGC CGA AGT CCT TT |
|  | reverse | TTG GAA CCG GCA TCT TTA TC |
| Gapdh | forward | AGC CCA AGA TGC CCT TCA GT |
|  | reverse | CCG TGT TCC TAC CCC CAA TG |

Supplementary Table 2. Antibody list used for IHC and Western blot in this study.

| Antibody | Host | Dilution | Application | source | catalogue number |
| --- | --- | --- | --- | --- | --- |
| MMP3 | Rabbit | 1:1000 | WB | Abcam,  Cambridge, UK | ab52915 |
| MMP13 | Rabbit | 1:1000 | WB | Abcam,  Cambridge, UK | ab51072 |
| ADAMTS5 | Rabbit | 1:250 | WB | Abcam,  Cambridge, UK | ab41037 |
| IL-1β | Mouse | 1:1000 | WB | Abcam,  Cambridge, UK | ab234437 |
| Tnf-α | Rabbit | 1:1000 | WB | Cell Signaling Technology, Beverly, MA | 3707S |
| IL-6 | Rabbit | 1:1000 | WB | Cell Signaling Technology, Beverly, MA | 12153S |
| IL-10 | Mouse | 1:1000 | WB | Abcam,  Cambridge, UK | ab33471 |
| p-PKA | Rabbit | 1:1000 | WB | Thermo Fisher Scientific, Waltham, MA | PA5-64489 |
| PKA | Rabbit | 1:1000 | WB | Thermo Fisher Scientific, Waltham, MA | PA5-17626 |
| p-CREB | Rabbit | 1:1000 | WB | Cell Signaling Technology, Beverly, MA | 9198S |
| CREB | Rabbit | 1:1000 | WB | Cell Signaling Technology, Beverly, MA | 9197S |
| p-AMPK | Rabbit | 1:1000 | WB | Thermo Fisher Scientific, Waltham, MA | PA5-17831 |
| AMPK | Mouse | 1:1000 | WB | Thermo Fisher Scientific, Waltham, MA | MA5-15815 |
| p-p38 | Rabbit | 1:1000 | WB | Cell Signaling Technology, Beverly, MA | 4631S |
| p38 | Rabbit | 1:1000 | WB | Cell Signaling Technology, Beverly, MA | 9212S |
| p-ERK1/2 | Mouse | 1:1000 | WB | Thermo Fisher Scientific, Waltham, MA | 14-9109-82 |
| ERK1/2 | Mouse | 1:1000 | WB | Thermo Fisher Scientific, Waltham, MA | 13-6200 |
| p-IκB | Mouse | 1:1000 | WB | Cell Signaling Technology, Beverly, MA | 9246S |
| IκB | Mouse | 1:1000 | WB | Cell Signaling Technology, Beverly, MA | 4814S |
| p-p65 | Rabbit | 1:1000 | WB | Cell Signaling Technology, Beverly, MA | 8242S |
| p65 | Rabbit | 1:1000 | WB | Cell Signaling Technology, Beverly, MA | 3033S |
| p-FOXO3a | Rabbit | 1:1000 | WB | Cell Signaling Technology, Beverly, MA | 9466S |
| FOXO3a | Rabbit | 1:1000 | WB | Cell Signaling Technology, Beverly, MA | 2497S |
| 8-oxo-dG | Mouse | 1:200 | IHC | Santa Cruz Biotechnology, Santa Cruz, CA | sc-66036 |
| Sirt1 | Rabbit | 1:1000 | WB | Cell Signaling Technology, Beverly, MA | 9475S |
| Nrf2 | Rabbit | 1:1000 | WB | Cell Signaling Technology, Beverly, MA | 12721S |
| HO-1 | Rabbit | 1:1000 | WB | Cell Signaling Technology, Beverly, MA | 70081S |
| β-actin | Mouse | 1:1000 | WB | Santa Cruz Biotechnology, Santa Cruz, CA | sc-47778 |
| HPR-linked  anti-rabbit | Donkey | 1:2000 | WB | Cell Signaling Technology, Beverly, MA | 7074s |
| HPR-linked  anti-mouse | Donkey | 1:2000 | WB | Cell Signaling Technology, Beverly, MA | 7076s |
| FITC-conjugated  anti-mouse | Donkey | 1:200 | IHC | Jackson Immuno Research Laboratories | 715-095-151 |
| IHC: immunohistochemistry; WB: Western blot | | | | | |

Supplementary table 3. Top 30 sequences that most effectively reduced LPS-induced NO production at 10 nM among 482 oligonucleotides

| Oligo_number | Sequence | NO change (%) compared to 1 µg/mL LPS treatment |
| --- | --- | --- |
| 359 | AGGGAGGGAGGGAGGGAT | -73.3272 |
| 281 | AGGGAGGGAGGGTG | -67.2976 |
| 260 | AGAGAGGAGAGGAG | -64.5636 |
| 259 | GAGAGAGAGAGAGA | -61.409 |
| 296 | GGAGGGAGGGAGGGA | -60.2524 |
| 283 | AGGGAGGGCGGGAG | -59.306 |
| 196 | AAGGAGGAGAGG | -57.0978 |
| 3 | AGGAGA | -56.1965 |
| 282 | AGGGAGGGTGGGAG | -53.7329 |
| 280 | GGGAGGGAGGCAGG | -53.4175 |
| 236 | AGAGAAGGAGGAA | -53.3123 |
| 241 | AAGAGAGGAGGAG | -53.2072 |
| 194 | GAGAGAGAGAGA | -52.6814 |
| 242 | AAGGGAGGGAGGG | -52.2608 |
| 235 | GGAGGAGAGGAGA | -51.3144 |
| 195 | AGAAGAGGAGGA | -50.2629 |
| 295 | GGAGGAGGAGGAGGA | -50.1577 |
| 233 | GAGAGAGAGAGGG | -49.3165 |
| 9 | AGAGAG | -49.1412 |
| 279 | GAGGCAGGAGGAGG | -49.1062 |
| 327 | AGAGGGAGGAGAGAGA | -48.6856 |
| 225 | GGAGAGGGTGA_G | -48.5804 |
| 326 | GAGAGAGAGAGAGAGG | -48.265 |
| 198 | AAGAGGAGAGGA | -47.9495 |
| 320 | AAGGGAGGGAGGGAGG | -47.7392 |
| 330 | GAGGGAGGGAGAGGGA | -47.3186 |
| 455 | GAGGAGGGAGGGAGGGAGGG | -47.2246 |
| 353 | AGGGAGGGAGCGGAGG | -47.0072 |
| 262 | AAGGGAAGGGAAGG | -47.0032 |
| 319 | GAGGAGGGAGGGAGGA | -46.5825 |

Supplementary table 4. Bottom 30 sequences that least reduced LPS-induced NO production at 10 nM among 482 oligonucleotides

| Oligo_number | Sequence | NO change (%) compared to 1 µg/mL LPS treatment |
| --- | --- | --- |
| 378 | GAGGAGGAGGAGGAGGA | 22.9434 |
| 408 | AGAAGAGAAGAGAAGAGA | 22.8890 |
| 154 | GGAGCGGAGG | 22.1314 |
| 404 | AAGGAAGGAAGGAAGGAG | 18.1605 |
| 106 | GGGAAGGGA | 17.92388 |
| 415 | AGGGAGGGAGGGAGGGAT | 17.8344 |
| 156 | GGAGAGCGGG | 17.58794 |
| 164 | GGAGGAGGAGG | 15.39614 |
| 407 | AGAAAGAAAGAAAGAAGG | 14.02974 |
| 144 | AGAGTAGGAG | 13.8603 |
| 406 | AAAGAAAGAAAGAAAGAA | 13.4862 |
| 374 | AGAGGAGGAGGAGGAGA | 12.9971 |
| 168 | AGGGAGAGGGA | 12.9324 |
| 146 | ACGAGAGGAG | 12.1965 |
| 128 | GAGAGAGAGG | 11.8445 |
| 118 | AGGGAGTGG | 11.8445 |
| 85 | GAGGATGG | 11.4445 |
| 416 | CAGGGAGGGAGGGAGGGG | 11.2578 |
| 148 | GGAGACAGAG | 10.2447 |
| 121 | GAGGTAGAG | 10.0847 |
| 111 | TGAGGGAGA | 10.0847 |
| 405 | GAGGAAGGAAGGAAGGAG | 10.0077 |
| 464 | TGGAGGGAGGGAGGGAGGGA | 9.9534 |
| 110 | AGAGGGAGC | 9.8127 |
| 145 | GAAGATGAAG | 9.76471 |
| 95 | GGAGGAGGA | 9.02879 |
| 149 | AGAAGGTGGA | 9.01279 |
| 180 | AGAGAAGGAGC | 8.8328 |
| 376 | AGGAAGGAAGGAAGGAG | 8.5946 |
| 113 | GTAGGAGAG | 8.51686 |

Supplementary table 5. Encapsulation efficiency (EE%) for oligonucleotide loading into PLGA nanoparticles using 1000 µg total input in a 4 mL batch

| No | Sample ID | Nucleic Acid Conc.( ng/µl) | A260 | A280 | 260/280 | 260/230 | EE(%) |
| --- | --- | --- | --- | --- | --- | --- | --- |
| 1 | supertant (Free oligo) | 147.7 | 5.083 | 2.729 | 1.86 | 1.83 | 40.92 |
| 2 | supertant (Free oligo) | 158.7 | 5.461 | 2.896 | 1.89 | 1.97 | 36.52 |
| 3 | supertant (Free oligo) | 166.4 | 5.727 | 3.038 | 1.89 | 1.99 | 33.44 |

**SUPPLEMENTARY FIGURE**

Supplementary Figure 1. Protective effects of NanoOligo on cartilage degradation and osteophyte formation in the DMM-induced mouse OA model. (A) Representative results of Safranin-O staining (N = 7). (B) Quantification of total OARSI grade (0-24), medial tibial plateau OARSI grade (0-6), subchondral bone plate thickness (μm²), and osteophyte size (mm²). *p < 0.05, **p < 0.01 compared to PBS-treated control DMM mice, Mann-Whitney U test.


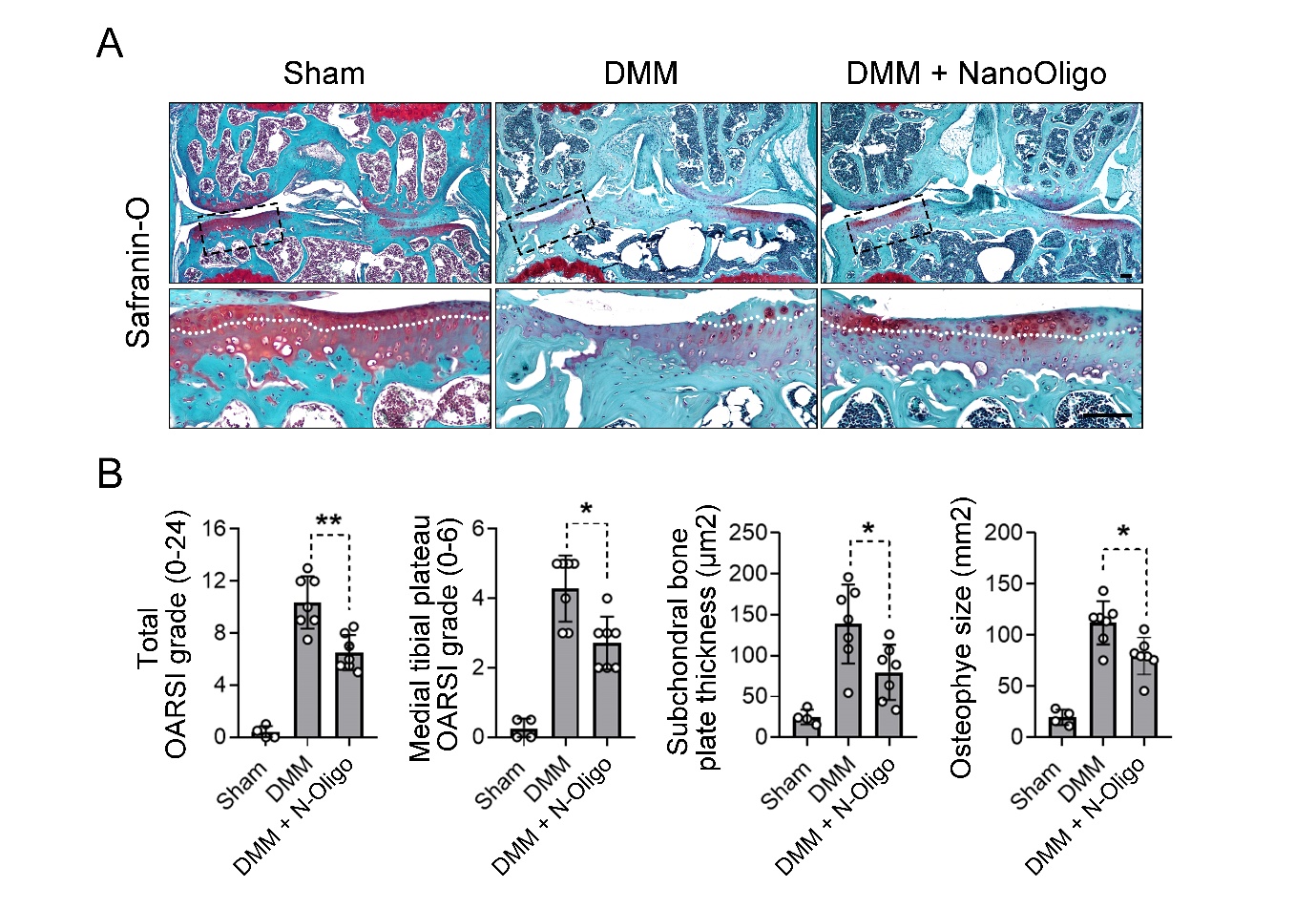


Supplementary Figure 2. Full blot image of Figure 3B.


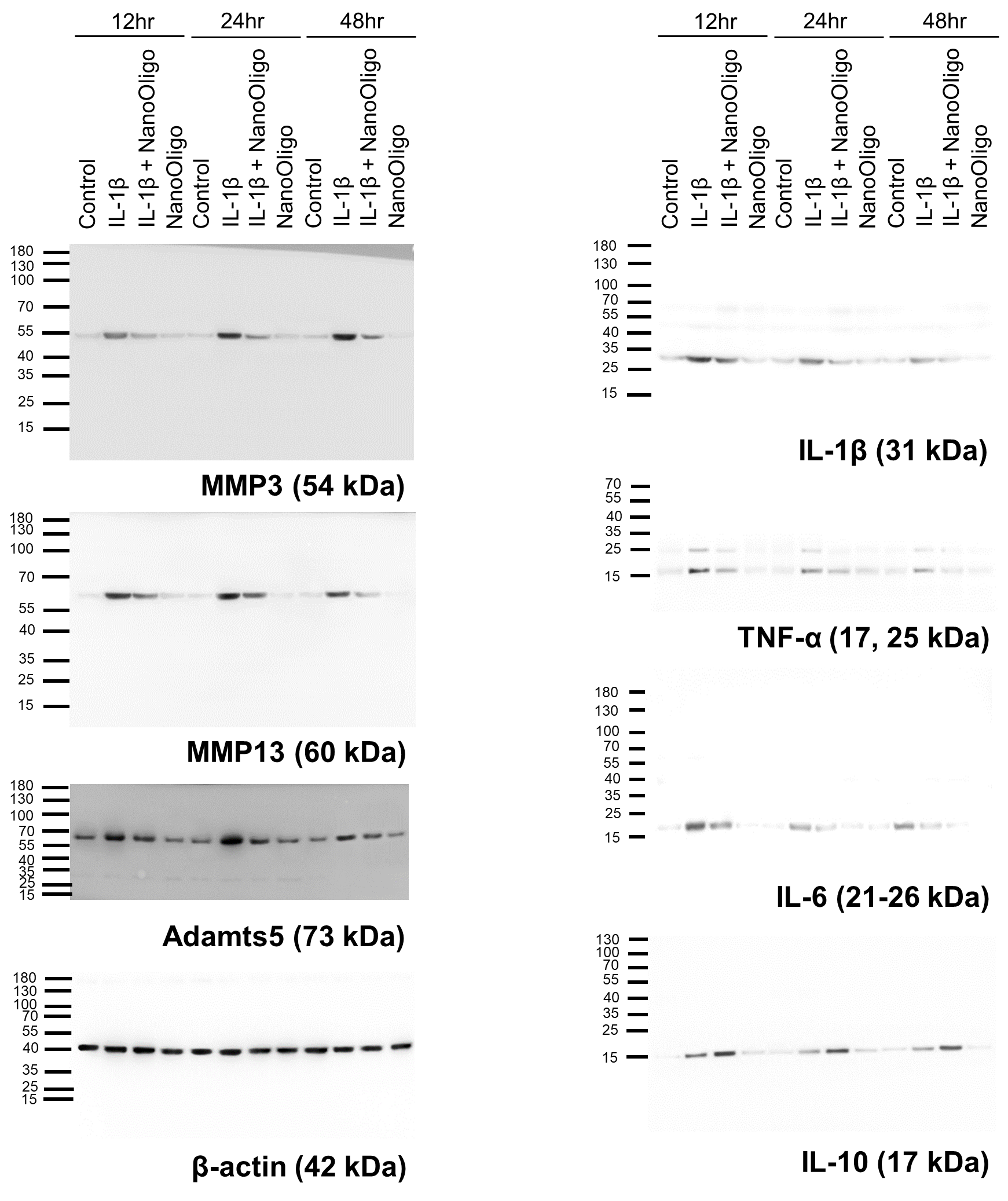


Supplementary Figure 3. Full blot image of Figure 3D


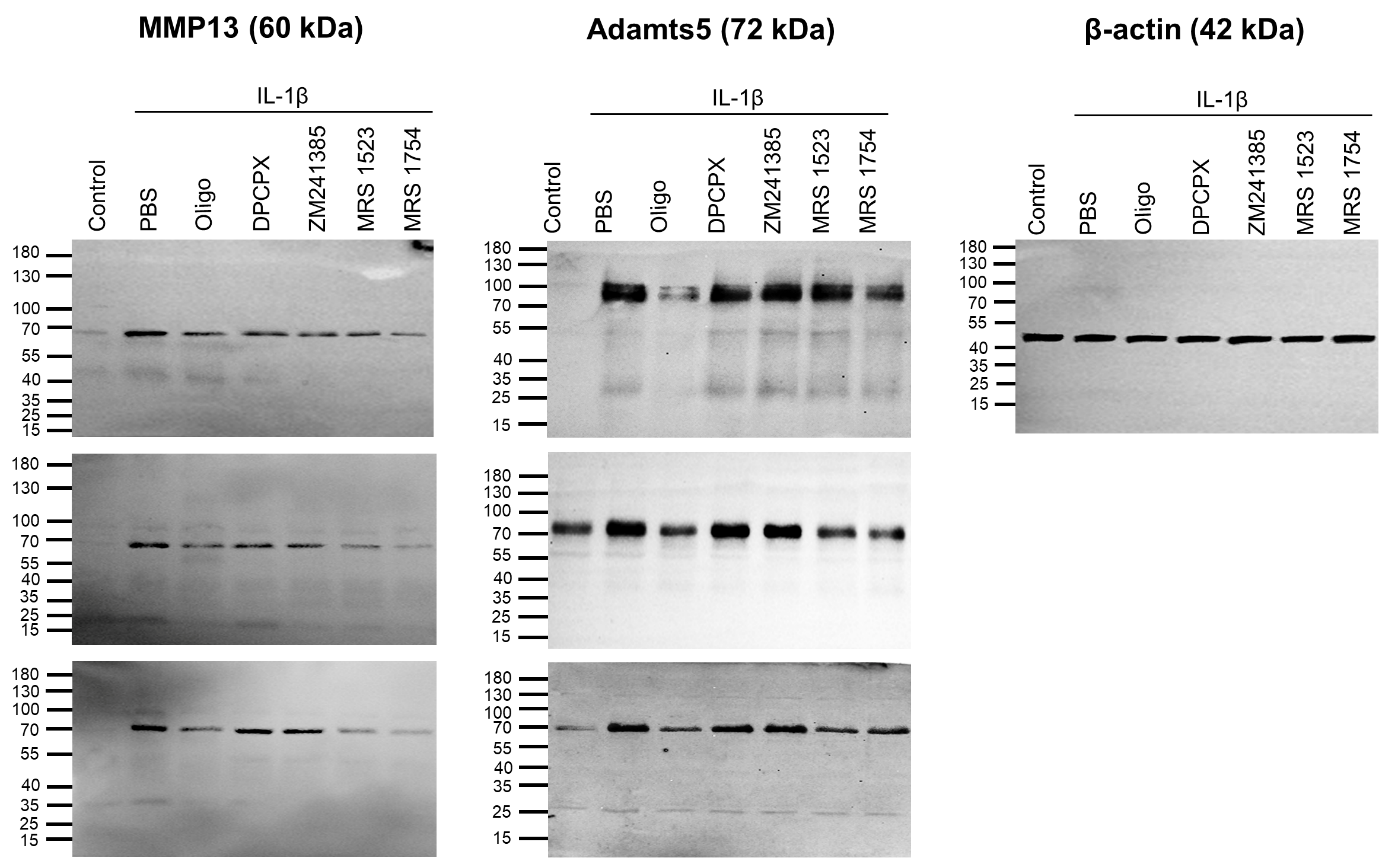


Supplementary Figure 4. Full blot image of Figure 4A


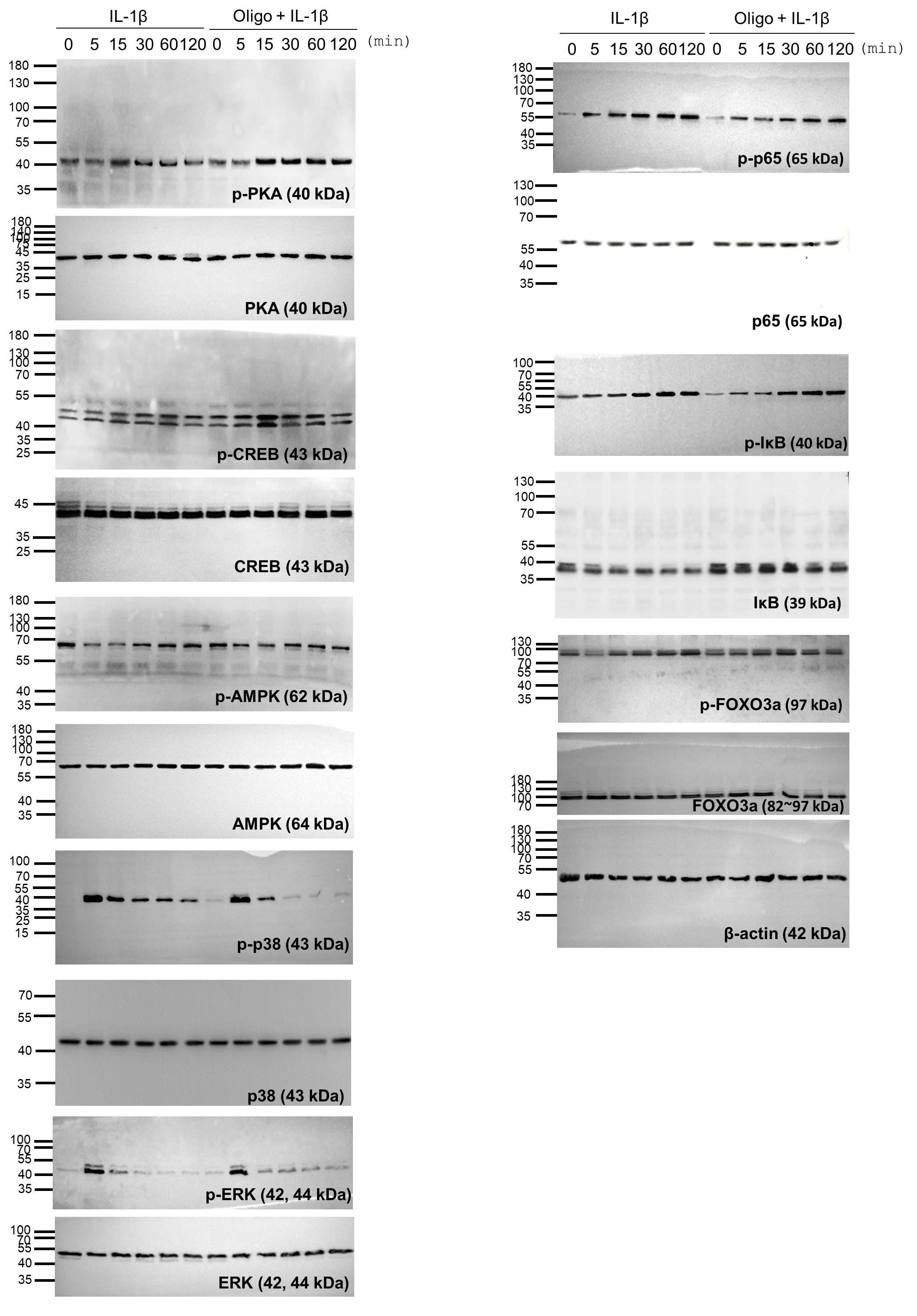


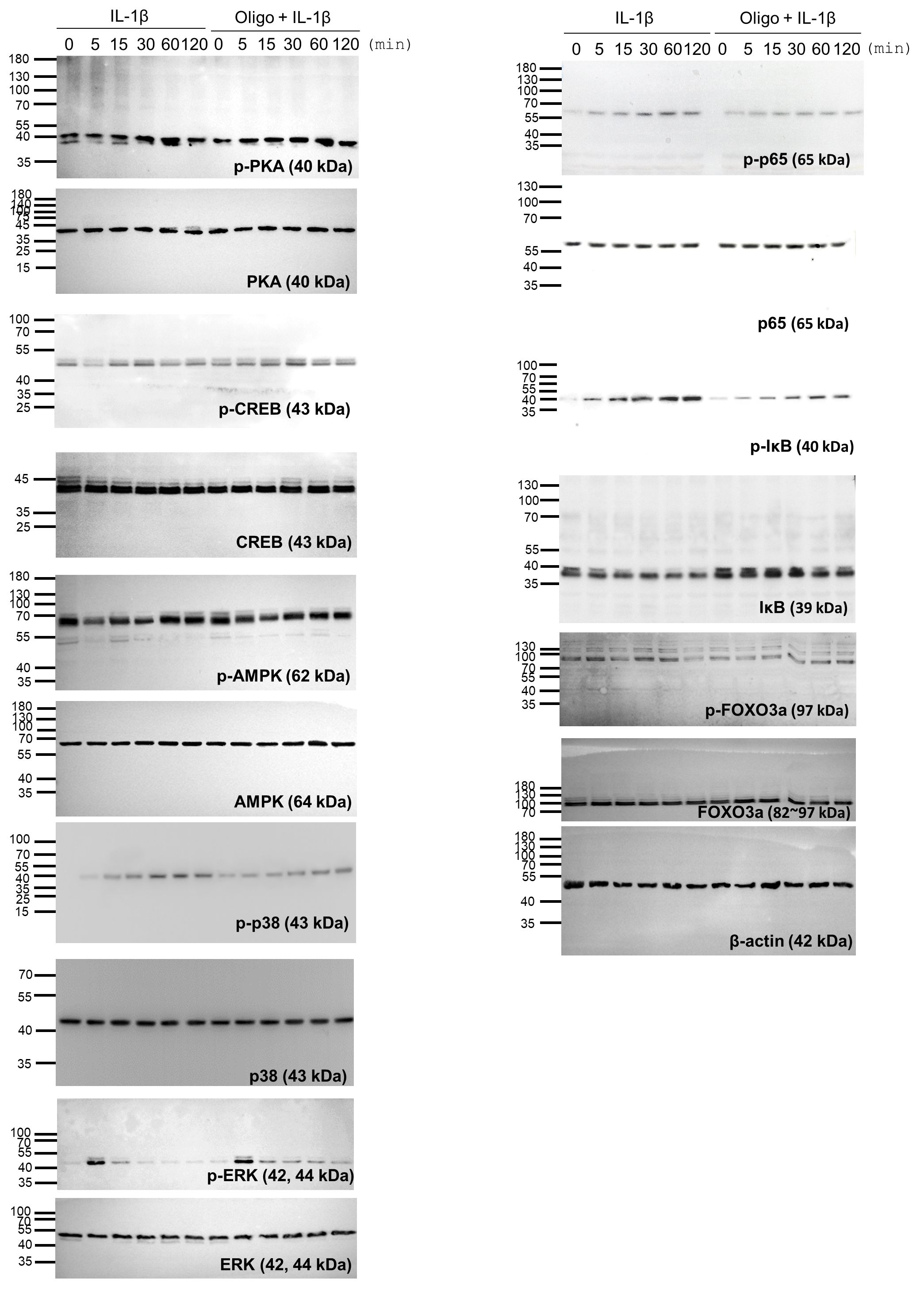


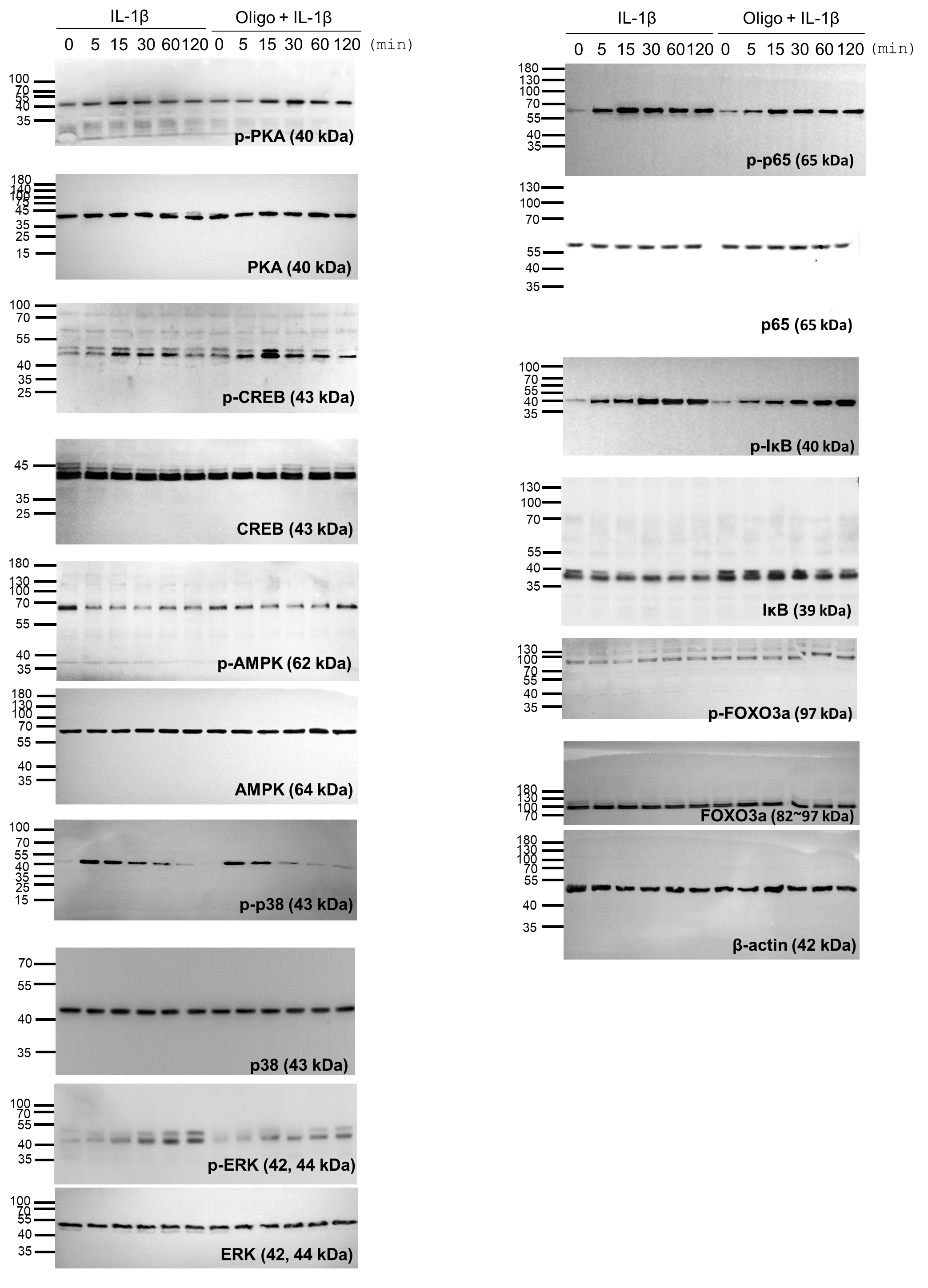


Supplementary Figure 5. Full blot image of Figure 5B


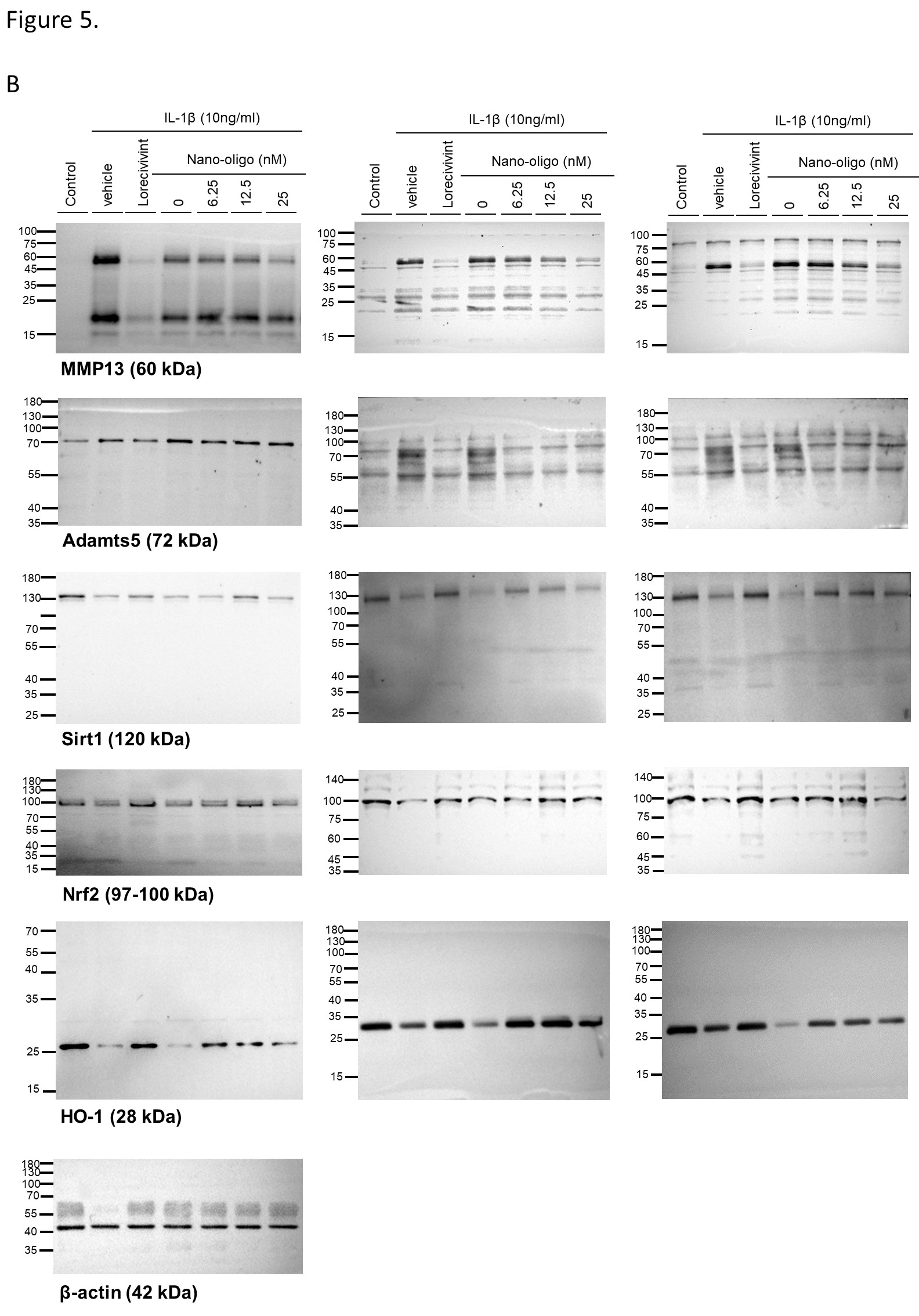

Supplement: Supplementary file 1 — Supplementary Material 1 [file 13346_2025_2020_MOESM1_ESM.docx]
